# D‐Peptide and D‐Protein Technology: Recent Advances, Challenges, and Opportunities**

**DOI:** 10.1002/cbic.202200537

**Published:** 2022-11-16

**Authors:** Alexander J. Lander, Yi Jin, Louis Y. P. Luk

**Affiliations:** ^1^ School of Chemistry Cardiff University Main Building, Park Place Cardiff CF10 3AT UK; ^2^ Manchester Institute of Biotechnology The University of Manchester Manchester M1 7DN UK

**Keywords:** chemical protein synthesis, chemical ligation, D-proteins, mirror-image proteins, peptides, proteins, protein engineering

## Abstract

Total chemical protein synthesis provides access to entire D‐protein enantiomers enabling unique applications in molecular biology, structural biology, and bioactive compound discovery. Key enzymes involved in the central dogma of molecular biology have been prepared in their D‐enantiomeric forms facilitating the development of mirror‐image life. Crystallization of a racemic mixture of L‐ and D‐protein enantiomers provides access to high‐resolution X‐ray structures of polypeptides. Additionally, D‐enantiomers of protein drug targets can be used in mirror‐image phage display allowing discovery of non‐proteolytic D‐peptide ligands as lead candidates. This review discusses the unique applications of D‐proteins including the synthetic challenges and opportunities.

## Graphical abstract

**D‐protein enantiomers** can be accessed through total chemical synthesis and their preparation enables establishment of mirror‐image life, structural analysis through racemic protein crystallography and discovery of proteolysis‐resistant ligands. Here, we highlight the recent advances in D‐protein and D‐peptide technology and discuss upcoming challenges and opportunities.

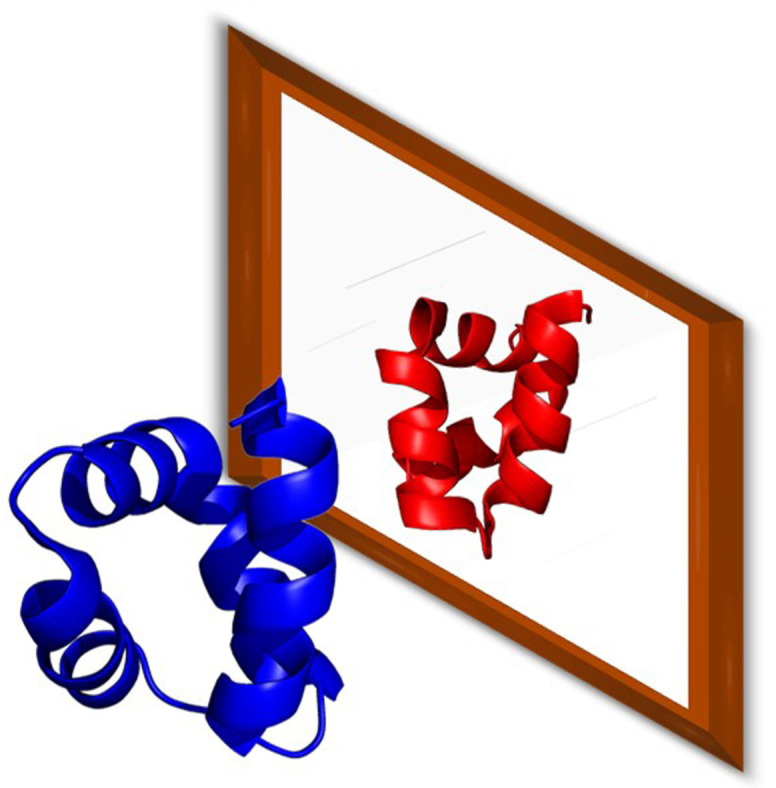

## 1 Introduction

**Figure 1:**
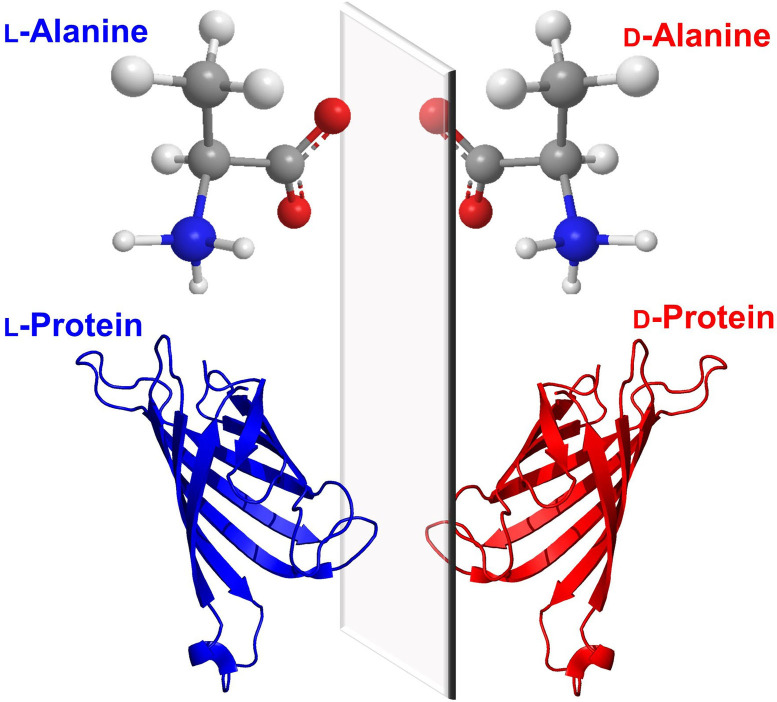
Proteins comprised entirely of D‐amino acids fold into the mirror image of the corresponding L‐protein.

Proteins, like other biomolecules, are composed of chiral building blocks.^[1]^ Ribosomes recruit L‐ (Levorotatory) amino‐acids for catalysis, and hence recombinant proteins are largely refrained in the L‐framework unless engineered ribosomes are used.^[2]^ In contrast, incorporation of D‐ (Dextrorotatory) amino acids into L‐polypeptides requires the use of engineered ribosomes,[^2a^, ^2b^] post‐translational modification systems (PLP‐dependent enzyme)^[3]^ or non‐ribosomal peptide synthetases (NPRS).^[4]^ At the time of writing, proteins entirely comprised of D‐amino acids have yet to be found in nature and must be synthesized via chemical routes. Though being more difficult to prepare, D‐amino acid proteins that fold into reciprocal chirality (Figure 1) possess extraordinary potentials in scientific research, spanning from the creation of mirror‐image life, mechanistic investigations of natural proteins to the isolation of ultra‐stable binders.^[5]^ In this review, we will describe current research surrounding enantiomeric proteins including both the challenges and opportunities.

## 2 Research Surrounding D‐Protein Enantiomers

### 2.1 Towards ‘mirror‐image life’

**Figure 2:**
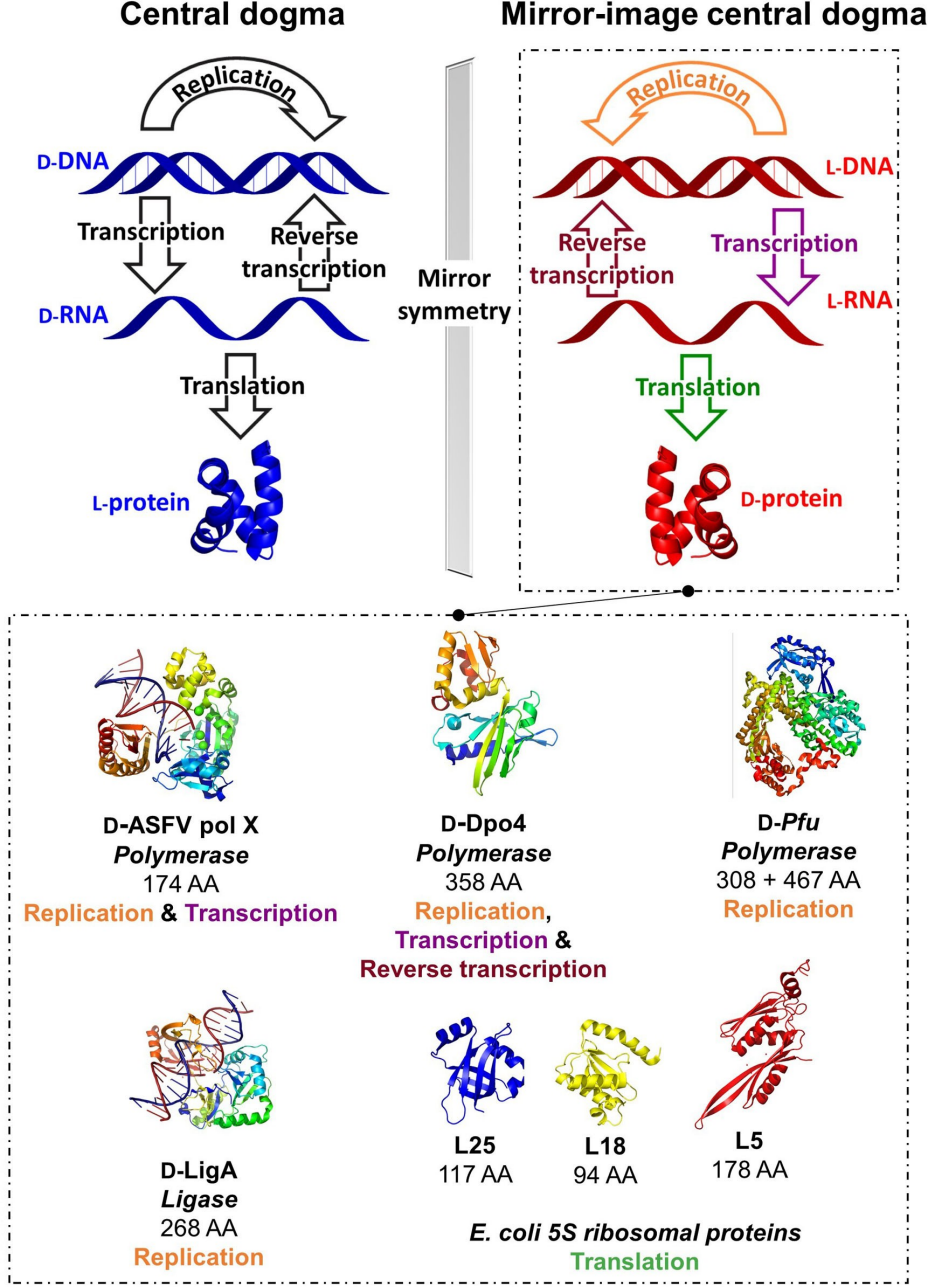
Recent advances in realizing mirror‐image synthetic biology using D‐protein enantiomers. Representative structures for illustrative purposes as D‐ASFV pol X (PDB: 1JQE), D‐Dpo4 (PDB: 3PR4), D‐Pfu (PDB: 2JGU), D‐LigA (PDB: 2Q2T), L5, L18 and L25 (PDB: 4YBB) constructed in PyMOL.^[11]^

Mirror‐image life which was first proposed by Louis Pasteur in 1860^[6]^ refers to the creation of an artificial biosystem with all macromolecules presented in their opposite enantiomeric forms. In these self‐replicating systems, L‐nucleic acids serve to store genetic information creating D‐protein workforce for biological function following the mirror‐image central dogma.^[7]^ Indeed. the *de novo* design of living entities has gained significant attention because of our fundamental interest in understanding the origin of life.^[8]^ While preparation of an entirely self‐replicating living entity in mirror image form is a major challenge, D‐enantiomers of key enzymes involved in the central dogma of molecular biology have been prepared (Figure 2).^[5]^ These enantiomeric enzymes hold potentials in research. For example, mirror‐image polymerases can be used to generate L‐nucleotide aptamer libraries^[9]^ or L‐genome for bioorthogonal information storage.^[10]^ Also, an enantiomeric ribosome could facilitate access to D‐proteins through mirror‐image translation. Furthermore, creation of a self‐replicating mirror‐image entity can offer access to D‐proteins, L‐sugars and/or other enantiopure pharmaceutical compounds. Recent examples of preparing D‐enzymes towards mirror‐image life are summarized below:

#### 2.1.1 Creation of artificial L‐polynucleotides by use of mirror‐image nucleic acid polymerases

Template‐directed polymerizations of the enantiomeric L–DNA and ‐RNA were first carried out using the D‐protein enantiomer of African swine fever virus polymerase X (D‐ASFV pol X).^[12]^ Composed of only 174 residues, ASFV pol X is the smallest DNA polymerase known, hence an ideal candidate for total chemical synthesis. The longest polynucleotide successfully replicated by D‐ASFV pol x was 44 nucleotides in length but required fresh enzymes at each cycle due to its weak thermal stability.^[12]^ A significant advancement was achieved by preparing the 358‐residue enantiomeric P2 DNA polymerase IV (Dpo4) from *Saccharolobus solfataricus*, which is sufficiently stable to temperature flux and could perform mirror‐image polymerase chain reaction (miPCR).[^9^, ^13^] The D‐Dpo4 could successfully create a 120‐bp L–DNA sequence encoding the *E. coli* 5S ribosomal RNA gene rrfB.^[9]^ Its thermostable variant D‐Dpo4‐3C could assemble a full L–DNA gene encoding protein Ssoo7d.^[13]^ Interestingly, a further‐engineered variant D‐Dpo4‐5m‐Y12S was reported that was capable of both transcription and reverse transcription, laying a strong foundation for enabling mirror‐image life.^[14]^

In order to create a lengthy enantiomeric gene with high fidelity, access to polymerase enzymes with a low error rate is essential, thus an enantiomeric derivative of *Pyrococcus furiosus* (Pfu) DNA polymerase has been deduced.^[15]^ Pfu is composed of 775 amino acids reaching 90 kDa in molecular weight, rendering its chemical synthesis challenging. To circumvent this issue, a split version of Pfu was prepared consisting of N‐ (467‐residue) and C‐ (308‐residue) fragments. Due to the significant cost of D‐isoleucine and its association with the aggregation of peptide fragments, most of them were replaced by other bulky residues including valine and leucine.^[10]^ The split polymerase D‐Pfu could synthesize a 1.5‐kb mirror‐image gene from short, synthetic oligonucleotides. Interestingly, because of the inherent stability towards enzymatic cleavage, a trace amount of artificial L‐DNA preserved in water from a local pond remained amplifiable by D‐Pfu after one year, whereas D‐DNA could not be amplified by L‐Pfu after one day. This work is a clear leap forward in the pursuit of mirror‐image biology. In addition, the miPCR platform is potentially useful in molecular discovery programs generating nuclease‐resistant L‐nucleotide aptamers for critical drug targets.^[9]^

#### 2.1.2 Other life‐essential, mirror‐image proteins

Enantiomeric ligase is another critical enzyme that can be used to create long stretches of L‐DNA.^[16]^ Preparation of a mirror‐image ribosome is exceptionally challenging as it composes of multiple protein and nucleotide subunits.^[17]^ Currently, three out of the ∼50 enantiomeric *E. coli* ribosomal proteins have been reported, including L5, L18 and L25.^[18]^ The D‐ribosomal proteins were prepared with native post‐translational modifications and interacted specifically with L‐5S RNA to form a mirror‐image ribonucleoprotein complex. On the other hand, eukaryotic ribosomes consist of 79–80 proteins and four rRNAs,^[17]^ requiring approximately 200 non‐ribosomal factors for assembly.^[19]^ Both the ribosomal proteins and rRNA itself also bear post‐translational modifications,^[20]^ adding further challenges to their preparation.

#### 2.1.3 Remarks

The genome of the laboratory strain *E. coli* K12 encodes for approximately 4300 proteins,^[21]^ but only a small fraction of their enantiomeric counterparts have been reported.^[22]^ Many of these proteins bear intrinsic synthetic challenges, because of their size, post‐translational modifications and folding (see section 3). Construction of the necessary mirror‐image oligonucleotides is also a major challenge. Whilst the synthesis of relatively short oligonucleotides is possible from L‐xylose or L‐arabinose,^[23]^ the resulting oligonucleotides suffer from lower purity.[^9^, ^12^, ^13^] With the advent of the high fidelity D‐Pfu capable of assembling complex genes,^[10]^ one might argue that this challenge is within reach. Given the significant efforts, it is also expected that a mirror‐image ribosome from bacteria will soon be reported. In addition, mirror‐image tRNAs, tRNA synthetases, and translation factors will need to be prepared to enable the translation of L‐mRNA to D‐polypeptides. When made available, the mirror‐image translation system will be game‐changing in the landscape of enantiomeric protein synthesis. Finally, pursuit of a truly self‐replicating system will require an approach of devising a minimal cell and assembly of each essential component in mirror‐image form.

### 2.2 Racemic protein crystallography

**Figure 3:**
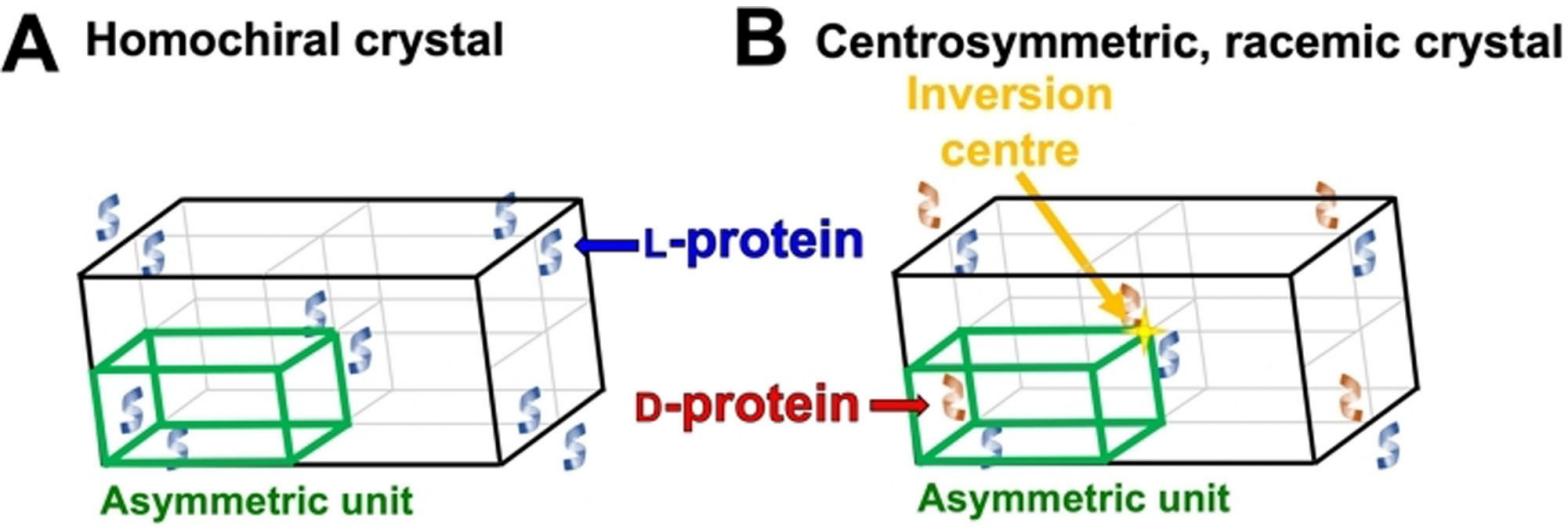
Illustrative comparison of (A) a homochiral crystal unit cell and (B) a racemic crystal unit cell containing an inversion center (e. g. *P* $\bar{1}$ ).

Racemic protein crystallography utilizes synthetic protein enantiomers for crystallization and is a technology particularly useful at yielding atomistic structural insights.^[24]^ Unlike native L‐proteins, racemic proteins can crystallize into achiral space groups possessing higher symmetry and order (Figure 3).^[25]^ Solving structure based on racemic proteins can be advantageous. As illustrated in the first example, the phase issue in solving the structure of rubredoxin was vastly simplified, because the space group was found to be centrosymmetric with the crystal unit cell containing a center of inversion (Figure 3B).^[26]^ The off‐diagonal phases of the X‐ray diffraction data obtained from a centrosymmetric crystal cancel out, restricting the phases to 0° or 180°, as opposed to the possible 0° to 360° arising from a homochiral crystal.^[24]^ Additionally, the centrosymmetric crystal has high dimensionality. This generally results in rapid protein crystallization and structure solving with high‐resolution detail, in addition to unveiling solute and ligand interactions.[^24^, ^27^]

Considering the higher order of crystal symmetry and favorable crystal growth, racemic protein crystallography has been used to resolve X‐ray structures of numerous proteins up to ultra‐high (sub‐angstrom) resolution (Table 1). Indeed, 13 (20 % so far) of the reported racemic crystal structures were resolved in the centrosymmetric space group *P* $\bar{1}$ . Many quasi‐racemic crystal structures, in which the enantiomers differ slightly, are reported to adopt the space group *P*1 due to a lack of true symmetry.^[28]^ However, it is more appropriate to classify these crystals as *pseudo‐*centrosymmetric or *“pseudo‐P* $\bar{1}$ .”[^25^, ^29^] While almost half of the racemic structures were resolved in centrosymmetric/pseudo‐centrosymmetric space groups, the other half were also resolved in chiral space groups (Table 1). Notably, there are no reported instances of a single enantiomer crystallizing into a chiral space group from a racemate. Thus, crystallization of proteins from racemates may have advantages beyond that explained by the achiral space group theory (for review – see ref. [24]).

**Table 1:** A decade of racemic protein crystallography (at the time of writing).

Protein	Function	Length^[a]^	Res. [Å]	Space group	PDB accession	Structural insights	Ref.
Lacticin Q	Bacteriocin	53	0.96	*P*1	7P5R	First reported crystal structure	[31]
CyO2	Bracelet cyclotide	30	1.17	*P*12_1_1	7RMQ	First reported crystal structures of bracelet cyclotides	[32]
CyO2 (I11L)			1.04	*P*12_1_1	7RMR		
CyO2 (I11G)			1.10	*P*12_1_1	7RMS		
Hyen D			1.35	*P*12_1_1	7RIH		
Hyen D (I11L)			1.22	*P* $\bar{1}$	7RII		
Hyen D (I11G)			1.30	*P*1	7RIJ		
Calcicludine	Kunitz‐type serine protease inhibitor homolog	60	2.52	*I*4_1_	6KZF	Confirmation of novel disulfide surrogate bridge strategy (DADA)	[33]
rC5a‐desArg	Rat anaphylatoxin	76	1.80	*P*1	^[g]^	New insights on C‐terminal conformation	[34]
Chimeric‐rC5a		77	1.31	*P*2_1_2_1_2_1_	^g^	Chimeric protein probes conjugated to small‐molecule antagonist	[35]
rC5a			1.58	*P* $\bar{1}$	^g^		
M2‐TM^[b]^	Ion channel TM helix	24	2.00	*P*2_1_ */c*	4RWB	Investigations of a heterochiral coiled coil	[27b]
M2‐TM^[c]^			1.05	*P* $\bar{1}$	4RWC		
M2‐TM (I39A)^[c]^			1.55	*P* $\bar{1}$	6MPL		[27a]
M2‐TM (I42A)^[c]^			1.40	*P* $\bar{1}$	6MPM		
M2‐TM (I42E)^[c]^			1.40	*P* $\bar{1}$	6MPN		
Melittin	Honeybee venom	27	1.27	*C*2	6O4M	Retention of native quaternary structure	[36]
Ribifolin	Orbitides from *Jatropha*	8	0.99	*P*12_1_/*n*1	6DKZ	Unveil structures of *Jatropha* orbitides	[37]
Pohlianin C		8	1.20	*Pcab*	6LD0		
Jatrophidin		8	1.03	P12_1_/*n*1	6DL1		
GsMTx4	Spider venom	34	1.75	*P* $\bar{3}$	^g^	First reported crystal structure	[38]
BTD‐2	Baboon θ‐defensin	18	1.45	*P* $\bar{1}$	5INZ	Novel oligomeric state resembles mechanistically relevant assembly	[39]
Snakin‐1	Potato snakin	63	1.50	*P*1	5E5Y	Novel use of radiation damage induced phasing of quasi‐racemic crystals	[40]
			1.60	*P*21/*c*	5E5Q		
			1.57	*P*21/*c*	5E5T		
Ubiquitin	Ubiquitin	76	1.95	^[d]^	^[g]^	Confirm folding of synthetic protein	[41]
M1‐linked tri‐Ubs		76^[e]^	1.80	*P*1	5GO7	D‐monomeric Ub can facilitate Ub oligomer crystallization	[28]
M1‐linked tetra‐Ub			2.18	*P*2_1_	5GO8		
K6‐linked di‐Ub			1.15	*P*1	5GOB		
K11‐linked di‐Ub			1.73	*P*1	5GOC		
K27‐linked di‐Ub			1.15	*P*1	5GOD		
K29‐linked di‐Ub			1.98	*P*2	5GOG		
K33‐linked di‐Ub			1.95	*P*1	5GOH		
K48‐linked di‐Ub			1.59	*P*1	5GOI		
K63‐linked di‐Ub			1.55	*P*2_1_2_1_2	5GOJ		
K11/K63‐linked tri‐Ub			1.84	*P*22_1_2_1_	5GOK		
K27‐linked di‐Ub		152	1.55	*C*2	5J8P	Largest true synthetic racemic proteins to be crystallized	[42]
K27‐linked tri‐Ub		228	2.10	*H*3	5JBV		
VHP	Vinillin headpiece domain	35	2.10	*P* $\bar{1}$	3TRW	Investigation of pentafluoro phenylalanine (F_5_Phe) amino acids on protein structure	[43]
VHP			2.30	*I*‐4*c*2	3TRY		
VHP (F_5_Phe10)			1.46	*F*222	3TJW		
VHP (F_5_Phe17)			1.00	*P*1	3TRV		
VHP (β3‐hGln26)			1.30	*P*1	5I1N	Investigation of beta amino acids on protein structure	[44]
VHP (ACPC26)			1.35	*P*1	5I1O		
VHP (β3‐hLys30)			1.40	*P*1	^[g]^		
VHP (APC30)			1.12	*P*1	5I1S		
Ts3	Scorpion venom	64	1.93	*P* $\bar{1}$	5CY0	First reported structure of Ts3	[45]
Magainin 2 (L‐1)	Amphibian HDP	23	1.75	*I‐*42*d*	4MGP	Beta amino acid variants investigating phenylalanine zipper motif	[46]
Magainin 2 (L‐2)			2.20	*P*2_1_2_1_2	5CGN		[47]
Magainin 2 (L‐3)			1.50	*P*1	5CGO		
ShK	Sea anemone venom	35	0.97	P12_1_/*c*	4LFS	Structure variation to NMR and enantiospecific activity	[48]
ShK analogue			1.20	*H*‐3	4Z7P	Structure activity relationships	[49]
ShK (allo‐Thr13)			0.90	*P*1	5I5B	Investigation of side chain chirality on protein structure	[50]
ShK (allo‐Thr31)			1.30	P12_1_1	5I5C		
ShK (allo‐Ile7)			1.20	*C*2	5I5A		
Rv1738	*M. tuberculosis* protein	94	1.50	*C*12/c1	4WPY	First reported structure, unknown function	[51]
STFI‐1	Sunflower trypsin inhibitor	14	1.25	*P* $\bar{3}$	4TTK	Disulfide‐rich scaffolds for drug design	[52]
cVc1.1	Cone snail venom	22	1.70	*Pbca*	4TTL		
kB1	Plant cyclotide	29	1.90	*P* $\bar{1}$	4TTM		
kB1 (G6A)			1.25	*P* $\bar{1}$	4TTN		
kB1(V25A)			2.30	*P* $\bar{1}$	4TTO		
Ser‐CCL1	Chemokine	73	2.15	*P*1	4OIJ	Crystal structure of a homogenous, glycosylated chemokine	[53]
Glycosylated Ser‐CCL1		73^[f]^	2.10	*P*1	4OIK		
DKP Ester Insulin	Synthetic hormone+derivatives	51	1.60	*P* $\bar{1}$	4IUZ	Confirm folding of synthetic derivative	[54]
Ester insulin			1.50	*I*2_1_3	5EN9	Confirmation of correctly folded synthetic protein for isotope experiments	[55]
Human insulin			1.35	*I*2_1_3	5ENA		
VEGF‐A/antagonist complex	Vascular endothelial growth factor A+D‐protein binder	102+56	1.60	*P*2_1_/n	4GLN	First reported structure of a heterochiral protein complex by racemic crystallography	[56]
Crambin analogue	Thionin protein	46	1.08	P12_1_1	3UE7	Novel linear‐loop peptide chain topology	[29a]
Kaliotoxin	Scorpion venom	38	0.95	*P* $\bar{1}$	3ODV	Basis for structure activity relationships	[57]
Omwaprin	Snake venom	50	1.30	*P*2_1_/*c*	3NGG	First reported structure	[30]

[a] Total amino acid length of synthetic protein enantiomer. [b] Crystallized from monoolein lipidic cubic phase. [c] Crystallized from racemic lipids. [d] Data unavailable. [e] Residues in D‐protein enantiomer. [f] 73 residues+oligosaccharide. [g] Not reported/deposited.

Racemic protein crystallography has been most frequently applied to study miniproteins containing fewer than 100 residues. These proteins are known to be difficult to crystallize because of their globular morphology which disfavors crystal packing. Meanwhile, their small sizes render the chemical synthesis of these proteins feasible (see section 3 below for synthetic approaches).^[30]^ Some of the unique insights generated by racemic crystallography are listed in Table 1.

#### 2.2.1 Quaternary states of protein

**Figure 4:**
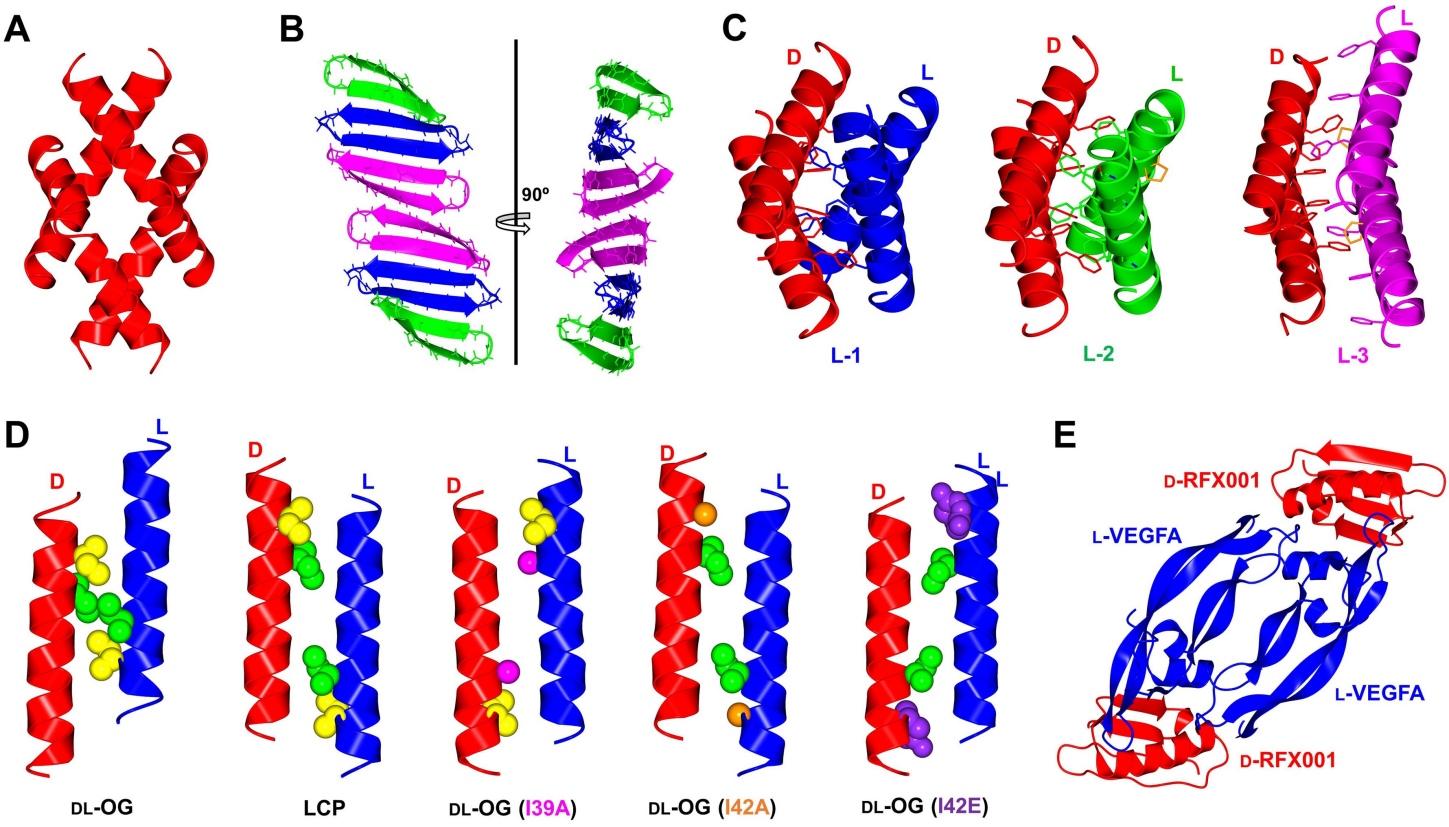
Quaternary structures by racemic protein crystallography; (A) Melittin tetramer (PDB: 6O4M); (B) BTD‐2 extended fibril‐like structure (PDB: 5INZ); (C) Magainin 2 phenylalanine zipper motif unaffected by β‐amino acid substitutions. D‐magainin 2 is shown in red and mutants L‐1 (Ala) shown in blue (PDB: 4MPG), L‐2 (APC) in green (PDB: 4CGN) and L‐3 (ACPC)(PDB: 5CGO) in magenta. β‐amino acids highlighted in orange; (D) M2‐TM helix forms heterochiral coiled coils, with a hendecad repeat identified in lipidic cubic phase (LCP, PDB: 4RWB) but absent in racemic β‐octylglucoside (DL‐OG, PDB: 4RWC). Mutation of sterically disruptive isoleucine residues to alanine (DL‐OG(I39A), PDB: 6MPL), (DL‐OG(I42A), PDB: 6MPM) or glutamate (DL‐OG(I42E), PDB: 6MPN) favored hendecad repeat motifs in chiral lipids; (E) Quaternary structure of VEGF‐A dimer bound to two D‐protein antagonist molecules (PDB: 4GLN). All structures were modelled in CCP4MG^[58]^ with data obtained from the Protein Data Bank.

Oligomeric assemblies are thought to play a common role in the activity of a variety of antimicrobial peptides, particularly those acting on the bacterial membrane.^[59]^ In an aim to elucidate its mechanism of action, the β‐sheet antimicrobial peptide originated from Baboons (BTD‐2) was chemically synthesized in both L‐ and D‐forms. Interestingly, racemic protein crystallography of BTD‐2 revealed a novel anti‐parallel trimeric form (Figure 4B). This supramolecular discovery is fibril‐like and is postulated to have critical roles in membrane disruption.^[39]^ In another example, melittin is an α‐helical antimicrobial peptide isolated from honeybee venom which is known to exert its activity by disrupting the bacterial cell membrane. The tetrameric assembly observed in the solution state is also present in the racemic X‐ray crystal structure (Figure 4A).^[36]^ Similarly, the tetrameric nature of magainin 2 was suggested to be critical for the activity of this amphibian host defense peptide (Figure 4C).^[47]^

Heterochiral interactions between L‐ and D‐protein isomers observed during structure elucidation may also lead to fruitful development in binder creation. In the studies of the transmembrane helix of the influenza M2 ion channel protein (TM‐M2),^[27b]^ a heterochiral coiled‐coil association was observed between the two peptide enantiomers in the presence of detergent octyl‐glucoside (DL‐OG) or within the monoolein lipid cubic phase (LCP) (Figure 4D). The LCP structure shows an 11‐residue helical repeat (hendecad, 3,4,4 spacing) in the coiled‐coil, which differs from homochiral coiled‐coils that adopts a 7‐residue helical repeat (heptad, 3,4 spacing). The crystals grown in DL‐OG do not form a hendecad repeat, as steric clashes involving Ile39 and Ile42 prevent proper 3,4,4 interaction. Substitution of these residues with alanine or glutamate produced the hendecad repeat coiled‐coil in the racemic DL‐OG structure, thus reinforcing the argument that hendecad repeats are a feature of heterochiral coiled coils.^[27a]^ Such heterochiral interactions can be used to design D‐proteins drugs, which are generally non‐proteolytic and non‐immunogenic (see section 2.3). The resulting drug‐target complexes can also be resolved using racemic protein crystallography to aid in rational optimization (Figure 4E).

#### 2.2.2 Post‐translationally modified proteins

**Figure 5:**
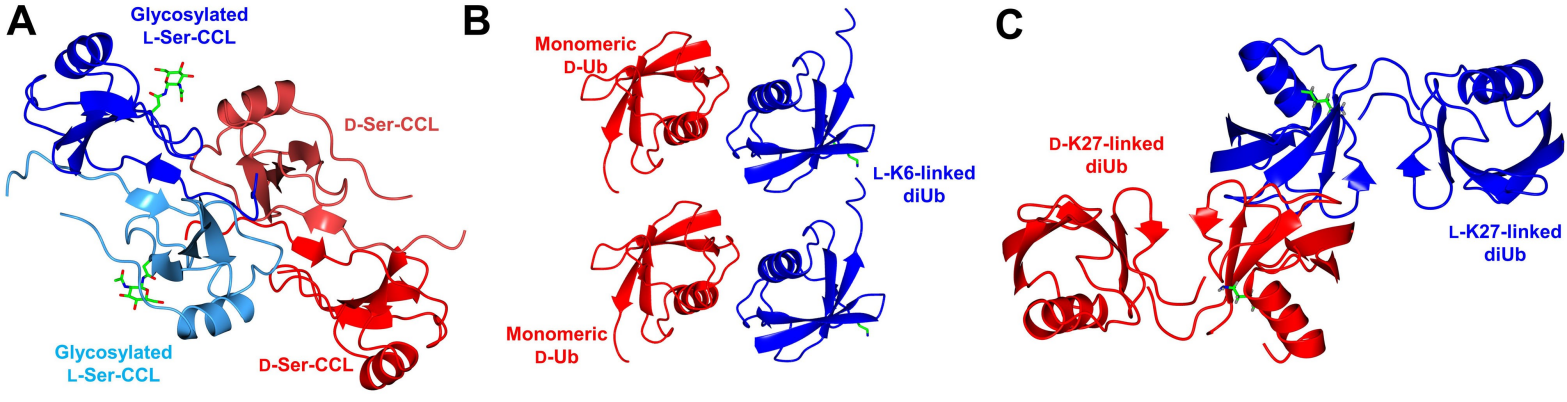
Quasi‐racemic protein crystallography of homogenous, post‐translationally modified proteins; (A) D‐Ser‐CCL facilitated crystallization of glycosylated L‐Ser‐CCL1 (PDB: 4OIK); (B) Monomeric D‐ubiquitin (D‐Ub) facilitated crystallization of L‐K6‐linked diUb (PDB: 5GOB); (C) Quasi‐racemic protein crystallography of L‐ and D‐K27‐linked diUb (PDB: 5J8P). All structures modelled in CCP4MG^[58]^ with data obtained from the protein data bank.

While obtaining homogenous, post‐translationally modified (PTM'd) proteins through recombinant methods remains a major technical challenge,^[60]^ chemical protein synthesis offers exquisite atomistic control and thus ensures homogeneity (see also section 3). Racemic protein crystallography of PTM'd proteins was first applied to the glycosylated chemokine Ser‐CCL1 protein, for which no structure was reported.^[53]^ The protein was synthesized in the native L‐form, followed by site‐specific asparagine *N*‐glycosylation with the native biantennary D‐glycan. Synthesis of the corresponding D‐enantiomer without glycosylation enabled the co‐crystallization of the quasi‐racemic protein (Figure 5A).

Another application involves the study of branched ubiquitin chains, where the folding of the branched protein molecules could be solved through racemic protein crystallography. Similarly, D‐ubiquitin was prepared in unmodified form^[41]^ and used to facilitate crystallization of the resulting branched L‐ubiquitin proteins (Figure 5B).^[28]^ A further example involved the preparation of branched ubiquitin proteins in both enantiomeric forms for racemic protein crystallography (Figure 5C), but the iso‐peptide linkages of the D‐proteins contained a non‐native cysteine residue scar to facilitate ligation.^[42]^

A key challenge, as presented in the former examples, is the preparation of PTM'd proteins in all D‐form. Glycosylated proteins possess glycans in native D‐chirality which would require complex synthesis from the corresponding L‐carbohydrates for a true racemic crystal. In addition, preparation of branched ubiquitin chains requires the use of non‐natural amino acids as auxiliaries for attachment of the ubiquitin, but they often suffer from poor ligation efficiency (see section 3.3.2.).^[42]^

#### 2.2.3 Remarks

Challenges associated with D‐protein synthesis, folding and PTMs hamper the application of racemic protein crystallography. The average size of a protein ranges from 283–438 residues in length^[61]^ with many bearing PTMs. Obtaining enough D‐protein for crystallization screening (generally in milligram range) remains labor‐intensive and uneconomical, typically requiring multiple chemical steps and protein refolding. Nevertheless, racemic protein crystallography of miniproteins remains an excellent method for deciphering molecular interactions, particularly serendipitous intermolecular interactions that deem difficult to obtain using homochiral protein crystallography, solution state NMR and/or computational structure prediction.[^27^, ^39^, ^44^, ^50^] Perhaps, a more promising avenue is to conduct quasi‐racemic crystallography where a minimal D‐protein is used to facilitate crystallization of a larger L‐protein with (pseudo‐)repeated domains.^[28]^

### 2.3 Identification of drug candidates through mirror‐image phage display and related screening technologies

**Figure 6:**
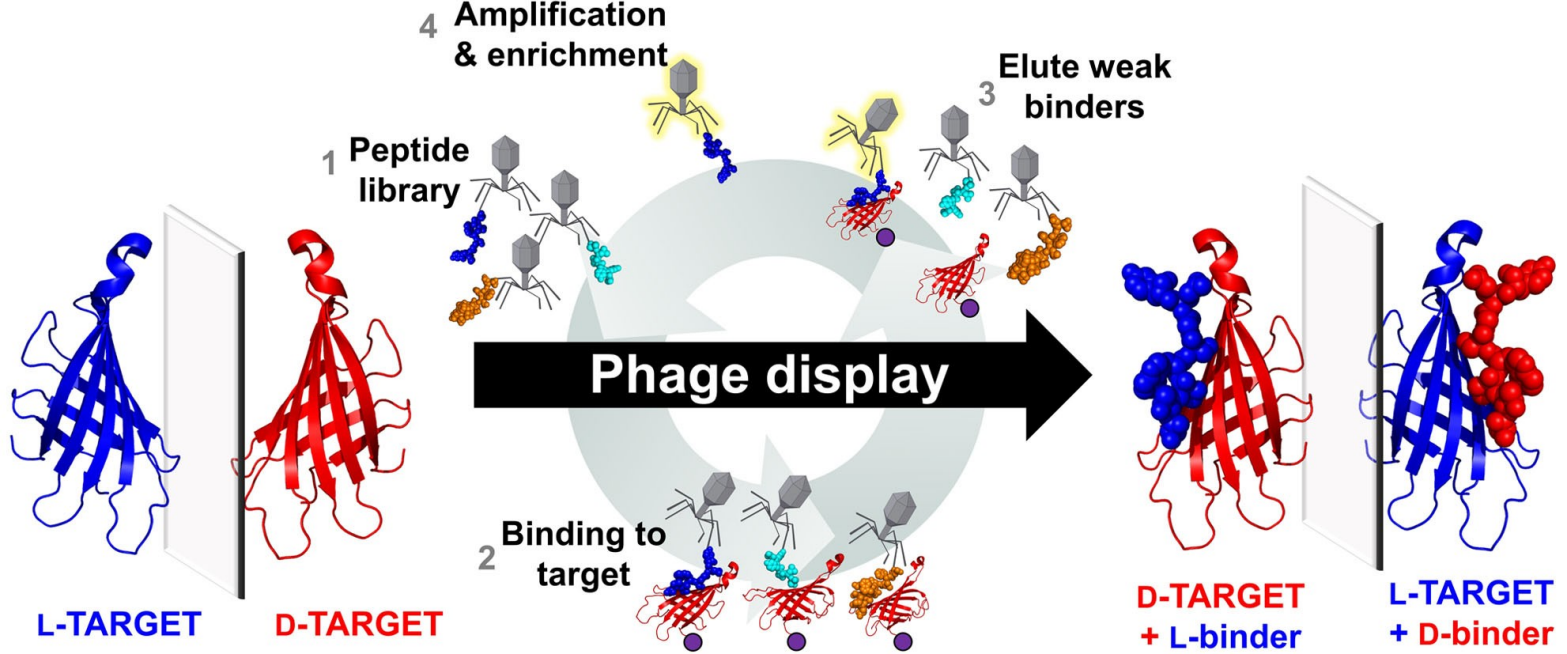
Process of mirror‐image phage display. Natural chirality L‐proteins are represented in blue, and mirror‐image synthetic D‐proteins are represented in red. Purple beads represent protein immobilization.

Polypeptide binders can offer significant selectivity and potency, and hence are excellent candidates for the treatment of various diseases and human disorders. One major bottleneck is that many peptide candidates suffer from proteolytic degradation, both limiting the option of the delivery methods and eliciting unwanted immune responses caused by major histocompatibility complex (MHC) presentation by immune cells.^[62]^ Peptides comprised of D‐amino acids are a viable approach as they are non‐recognizable by endogenous proteases.^[63]^ It has been suggested that D‐peptide binders can be made by retro‐inversion (RI) which relies on flipping the entire peptide chain from the N‐ to C‐ termini to offset the flip in the side chain chirality.^[64]^ Though some success has been seen in short binders, it was quickly discovered that this double‐flip approach did not reinstate the true peptide structure and, in some cases, could drastically weaken the peptide binding.^[65]^ Another approach was to use D‐peptides to mimic the shape of the L‐peptide agonist when presented in an MHC, without reference to the L‐peptide sequence, acting as a stable vaccine candidate.^[66]^ Recently, binders composed of both D‐ and L‐residues have been developed through ribosomal engineering^[67]^ or *in silico* protein design.^[68]^ In order to create binders entirely composed of D‐amino acids, the most routine approach is mirror‐image phage display (MIPD).^[69]^ In MIPD, D‐enantiomers of protein targets are synthesized and subjected to L‐peptide screening (Figure 6). Due to the nature of mirror‐image symmetry, the same‐sequence D‐peptide will bind to the native L‐protein target with equal affinity, thus yielding an inherently non‐proteolytic peptide binder. MIPD has discovered D‐peptide binders for a range of targets (Table 2). Key examples are summarized in Table 2.

**Table 2:** D‐peptide binders identified through mirror‐image phage display of D‐protein targets in the last decade (at the time of writing).

Protein	Function	Length^[b]^	Name	Type	Length^[c]^	K_d_ [μM]^[d]^	Ref.
ARQ23	Androgen receptor	46	QF2D‐2	Linear	16	11	^[81]^
Annexin A1 NTD	Surface marker in malignant tumor vasculature	16	D‐TIT7	Linear	7	8.5 x10^−3^	^[82]^
Tau PHF6*	Microtubule‐associated protein	6	MMD3	Linear	12	^**[e]**^	^[83]^
			MMD3rev	Linear	12	^**[e]**^	
Tau PHF6			p‐NH	Linear	12	^**[e]**^	^[80b]^
			TD28	Linear	12	^**[e]**^	^[80a]^
Immunoglobulin variable domain of TIGIT	Immune checkpoint	119	D‐TBP‐3	Linear	12	5.6	^[77b]^
Epidermal growth factor	Mitogenic factor	53	D‐PI_4	Linear	12	54	^[72]^
Fibroblast growth factor‐inducible 14 CRD	TWEAK (tumor necrosis factor‐like weak inducer of apoptosis) receptor	43	D‐FNB	Linear	12	0.28	^[84]^
Aβ‐1‐42	Monomeric precursor of AB oligomers and fibrils	42	Mosd1	Linear	12	^**[e]**^	^[85]^
Immunoglobulin variable domain of PD‐L1	Programmed cell death protein 1	124	D‐PPA‐1	Linear	12	0.51	^[77a]^
VEGF‐A	Vascular endothelial growth factor	102	RFX‐V1a2a	Bivalent scaffold	53+58	8 x10^−4^	^[75]^
			RFX001	GB1 scaffold	56	8.5 x10^−2^	^[56]^
gp41N‐trimer pocket mimic	HIV envelope protein ectodomain	42	PIE12‐trimer	Flanking disulfide cyclic	8^**[f]**^	^**[e]**^	^[78b]^
MDM2	Oncogenic E3 ubiquitin ligase	85	D‐PMIα	Linear	12	5.3 x 10^−2^	^[86]^

[a] Most potent binder from phage panning experiments presented. [b] Total amino acid length of synthetic protein enantiomer target. [c] Amino acid length of peptide binder identified through phage display. [d] Reported dissociation constant of D‐peptide binder to native L‐target used in phage display. [e] K_d_ not reported. [f] Length of original, un‐crosslinked peptide identified through phage display.

#### 2.3.1 D‐Peptides as potential anticancer lead candidates

Growth factor proteins and/or their receptors are overexpressed in many types of cancer,^[70]^ and hence development of their antagonists can hinder malignant tumor growth as a form of treatment in cancer therapy.^[71]^ Consequently, enantiomeric segments of both the epidermal growth factor (EGF) and vascular endothelial growth factor (VEGF‐A) were synthesized for MIPD.[^56^, ^72^] A 12‐residue linear D‐peptide ligand for EGF, D‐PI_4, was identified with both binding affinity and half‐maximal inhibitory concentration (IC_50_) in micromolar range.^[72]^ In the case of VEGF‐A, the mini‐protein GB1 was used as a template scaffold to create a 56 residue D‐mini‐protein RFX001.D that has a binding affinity as low as 85 nM.[^56^, ^73^] A heterochiral protein complex between a vascular endothelial growth factor (L‐VEGF‐A) and a D‐protein antagonist was also solved using racemic protein crystallography (Figure 4E), providing a foundation for structure‐based optimization of the D‐protein antagonist.^[56]^ Upon optimization, the binder RFX037.D was created, increasing both binding affinity (*K*_D_=6 nM vs 85 nM) and thermal stability (*T*_m_ >95 °C vs 33 °C).^[74]^ Of note, RFX037.D was non‐immunogenic in mice, whereas the L‐enantiomer generated a strong immune response. In an extension of the MIPD against VEGF‐A, bivalent D‐protein ligands were also developed using orthogonal MIPD assays with two different scaffold mini‐proteins (53 and 58 residues).^[75]^ The two best scaffolds were connected via a covalent linkage to yield the bivalent D‐protein RFX‐V1a2a, with sub‐nanomolar (*K*_D_=0.8 nM) affinity for VEGF‐A.

Other key targets for cancer treatment are immune checkpoints,^[76]^ which are often suppressed by cancer cells to avoid recognition by the innate immune system. The immunoglobulin‐like variable (IgV) domains are known to govern immune checkpoints, and thus enantiomeric counterparts of the IgV domains of the programmed‐cell death protein ligand 1 (PD‐L1, 124 residues) and the T‐cell immunoglobulin and ITIM domain (TGIT, 119 residues) were synthesized for MIPD.^[77]^ After five rounds of biopanning, binders with micromolar affinity and IC_50_ were achieved, presenting themselves as promising drug candidates.^[77a]^ One specific binder D‐TBP‐3 demonstrated proteolytic stability and, importantly, the ability to penetrate through tumor tissue in mice which resulted in tumor suppression.^[77b]^

#### 2.3.2 D‐peptides as lead preventive therapeutic candidates

The development of potent D‐peptide antagonists of the HIV‐1 envelope protein gp41 was shown to prevent viral fusion and entry into cells.^[78]^ A trimeric version of one of the isolated candidates could block pocket‐specific viral entry with an IC_50_ as low as 250 pM.^[78a]^ Further pharmacokinetic optimization and synthetic scale‐up yielded the cholesterol‐conjugated trimeric D‐peptide CPT31,^[79]^ which is currently in Phase Ia clinical trials for the treatment of HIV.

In another study, MIPD was used to create D‐peptides with high affinity for the microtubule‐binding protein Tau, preventing self‐aggregation in the treatment of tauopathies.^[80]^ The hexapeptides PHF6 (VQIINK) and PHF6* (VQIVYK) were found to promote Tau aggregation in tauopathies such as Alzheimer's disease, and their enantiomers have been used as a target for MIPD.[^80^, ^83^] Notably, two peptide candidates, MMD3 and MMD3rev, demonstrated cell‐penetrating properties, with the ability to cross the cell membrane of neurons.^[83]^

#### 2.3.3 Remarks

Despite all the research efforts, there is no D‐peptide therapeutic that has yet reached the market. To our knowledge, CPT31 is the only D‐peptide candidate that has entered early‐stage clinical trials, and the estimated success rate of bringing a binder from phase I to approval is 14 %.^[87]^ Discovery of D‐peptide binders remains challenging hampering downstream clinical research and product development. The ultimate challenge of MIPD lies within the preparation of the enantiomeric protein target. Not only can size be a concern, but both the PTM and protein folding status can also pose major synthetic challenges (see section 3 for synthetic approaches). Except for the Tau targeting peptides, many targets are restricted to extracellular protein domains, as cell‐penetrating properties of peptide binders are often weak. In addition, the use of MIPD to identify competitive antagonists is limited by the arbitrary selection of off‐target binders. Efforts have been directed to computation‐based approaches with the goal to replace the tedious synthesis with *in silico* studies, including the docking of mirror‐image helices derived from the PDB;^[88]^ screening of D‐tri/tetra peptides against the target active site;^[89]^ virtual affinity maturation based on existing heterochiral structures.^[90]^ However, existing *in silico* methods suffer from a lack of library diversity and polypeptide binder size, limiting the best example to a binding affinity of 20 μM.^[89]^

## 3 The Current State of the Art in D‐Polypeptide Preparation

The most common issue encountered in enantiomeric protein research surrounds their preparation. Since polypeptides entirely composed of D‐amino acids cannot be made recombinantly at the time of writing, they must be prepared by chemical synthesis and the current state‐of‐the‐art is summarized as below:

### 3.1 Solid phase peptide synthesis (SPPS)

Allowing stepwise addition of protected amino acids on an insoluble polymer support, solid phase peptide synthesis (SPPS) facilitates access to D‐polypeptide chains.^[91]^ Fmoc‐protected amino acids have gained popularity over the past two decades as they facilitate the use of milder cleavage conditions.^[92]^ Efficient reagents that allow high conversion of amino acid coupling have been reported.^[93]^ The systematic nature of SPPS has also led to automated systems.^[94]^ When paired with microwave irradiation, each amino acid coupling cycle can be performed in four minutes.^[95]^ Recently, a fully automated system for SPPS, where the assembly is complete in a flow system has been developed, yielding complete coupling cycles in less than two minutes, and a full protein up to 164 residues has been assembled.^[96]^ However, a major drawback is its requirement for a large excess of amino acids (6–60 equivalents), a major financial burden when it comes to D‐polypeptide synthesis. This is especially the case when the target proteins contain diastereomeric D‐isoleucine, which is significantly higher in cost than other building blocks.

### 3.2 Chemical ligation

**Figure 7:**
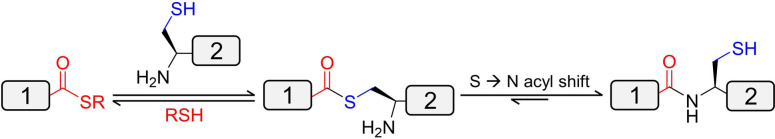
Reaction scheme of native chemical ligation.

To bring down the cost, convergent synthesis of proteins through the assembly of smaller polypeptides by chemical ligation has been achieved.^[97]^ In general, two peptide fragments are chemically bought together by reacting two latent reaction motifs located at the termini. Various methods for chemical protein ligation have been developed, although some suffer drawbacks when preparing polypeptides in opposite chirality due to requiring modified amino acids (Table 3, Entries 1–4). On the other hand, native chemical ligation (NCL) and serine‐threonine ligation (STL) employ canonical cysteine or serine/threonine residues, though the latter is yet to be reported in D‐protein synthesis. NCL is commonly used whereby the thiol group of an N‐terminal cysteine peptide and a C‐terminal thioester of a reacting pair undergo *trans*‐thioesterification, followed by an S‐to‐N acyl shift to yield a traceless native peptide bond (Figure 7).

**Table 3:** Synthetic methods used in chemical protein synthesis, indicating use in reported D‐protein synthesis and potential issues encountered with use.

Entry				Used in D‐protein synthesis	
#	Synthetic method	Reagents	**Y** / **N**	Potential issues	Ref.
	Ligation method				
		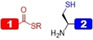			
1	Native chemical ligation	+ thiol catalyst	Y	Dependence on suitable cysteine or alanine residues.	[97a]
		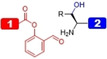			
2	Serine/threonine ligation	+ acidolysis	N	Requires suitable Ser/Thr. Slower reaction kinetics than NCL.	[145]
3	KAHA ligation	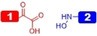	N	Accessibility of enantiomeric reagent.	[146]
		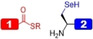			
4	Selenocysteine NCL	+ thiol catalyst	N	Accessibility of enantiomeric reagent.	[123a]
	NCL reactive end				
	Thioester surrogate				
		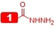			
5	Hydrazides	Activation+NaNO_2_ + Thiol	Y	Oxidation incompatible with Thz. Low temperature activation needed (<−15 °C).	[100]
		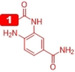			
6	Dbz	Activation 4‐nitrophenyl chloroformate or NaNO_2_ or isoamyl nitrite +thiol	Y	Di‐acylation side product with excess Gly.	[16, 147]
		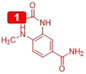			
7	MeDbz	Activation 4‐nitrophenyl chloroformate +thiol	N	Difficult to activate off‐resin.	[98]
		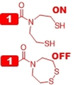			
8	SEA	Activation of SEA_OFF_ TCEP+thiol	N	Latent SEA_OFF_ thioester incompatible with TCEP during ligations.	[148]
	N‐cysteine protection				
		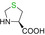			
9	Thz	Deprotection MeONH_2_	Y	Incompatible with hydrazide oxidation.	[103]
		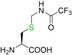			
10	Cys(Tfacm)	Deprotection pH 11.5	N	Accessibility of enantiomeric reagent.	[149]
		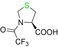			
11	TFA‐Thz	Deprotection Base then MeONH_2_	Y	Accessibility of enantiomeric reagent.	[41]
		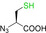			
12	N_3_‐Cys	Deprotection TCEP	N	Accessibility of enantiomeric reagent.	[150]
		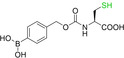			
13	Cys(Dobz)	Deprotection H_2_O_2_	N	Accessibility of enantiomeric reagent. Harsh deprotection conditions.	[151]
	Desulfurization				
14	Metal‐based	Pd/Al_2_O_3_ Or Raney Nickel+ H_2 (g)_	Y	Removal of metal impurities can be problematic. Use of hydrogen gas. Potential side reactions with Trp and Met Quenched by thiol catalyst. Native Cys must be protected.	[108]
15	Metal‐free radical based	VA‐044 TCEP *tert*‐butylthiol	Y	Quenched by thiol catalyst. Native Cys must be protected.	[106]
16	Beta/gamma thiol amino acids	β‐thiol‐Phe β‐thiol‐Val β‐thiol‐Leu β‐thiol‐Asp β‐thiol‐Asn β‐thiol‐Arg γ‐thiol‐Val γ‐thiol‐Thr γ‐thiol‐Ile γ‐thiol‐Pro γ‐thiol‐Glu γ‐thiol‐Gln γ‐thiol‐Lys 2‐thiol‐Trp	N	Accessibility of enantiomeric reagent. Commercially available D‐Penicillamine (β‐thiol‐Val) could be used for D‐peptide ligation at Val, if directly following a glycine residue.	[152]
	Thiol catalysts for one‐pot ligation‐desulfurization				
		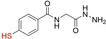			
17	MPAA‐hydrazide	pK_a_=6.6 Removal Aldehyde‐resin capture	N	Preparation of MPAA‐hydrazide reagent coupled with use in large excess is uneconomical.	[153]
		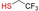			
18	Trifluoroethanthiol	pK_a_=7.3 Removal Evaporation (bp=37 °C)	N	Malodorous and volatile, though could be used for D‐protein synthesis.	[154]
		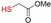			
19	Methyl thioglycolate	pK_a_=7.9 Removal none	Y	Slower kinetics with C‐terminal beta‐branched residue.	[41]
	Solubility enhancers				
		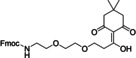			
20	Helping hand v1	Installation Amine labelling with lysine side chain Removal Hydrazine _(aq)_	N	Additional steps to incorporate and remove tag. Potential issues with stability.	[119a]
		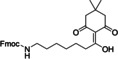			
21	Helping hand v2	Installation Amine labelling with lysine side chain Removal Hydrazine _(aq)_ Or Hydroxylamine _(aq)_	N	Additional steps to incorporate and remove tag.	[119b]
		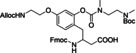			
22	Removable backbone modification v1	Installation Standard Fmoc‐SPPS Removal pH 7 then TFA	Y	Limited to Gly only. Lengthy synthesis of building block. Additional steps to incorporate and remove tag.	[117, 155]
		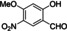			
23	Removable backbone modification v2	Installation Reductive amination Acetylation Removal Deacetylation (Cys _(aq)_) then TFA	Y	Additional steps to incorporate and remove tag.	[77b, 118]
	Protein folding				
		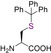			
		Disulfide #1 Removal TFA			
		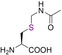			
		Disulfide #2 Removal Iodine or PdCl_2_			
		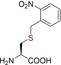			
24	Cysteine orthogonal protection	Disulfide #3 Removal UV light (350 nm)	Y	Practically limited to two disulfide bonds. Accessibility of a third, orthogonally protected D‐Cys building block.	[52, 128]
25	“Ambidextrous” chaperone	GroEL/ES protein chaperone	Y	Mostly unnecessary for *in‐vitro* protein folding. Limited scope reported.	[121b]
	Post‐translational modifications				
26	Lys ubiquitination	δ‐mercapto lysine for NCL followed by desulfurization	N	Accessibility of enantiomeric reagent.	[136a]
27		Solid‐phase isopeptide bond formation	N	Requires assembly of large fragments by SPPS – not cost‐effective with D‐amino acids	[156]
		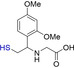			
28		Installation Coupling to lysine side chain PTM NCL to Ub‐thioester Auxiliary removal TFA	N	Low efficiency of ligation. Glycyl auxiliary replaced with Cys in preparation of enantiomeric di‐ and tri‐ubiquitin proteins.	[28, 42]
29	Lys trimethylation	Fmoc‐Lys(Me_3_)‐OH	N	Accessibility of enantiomeric reagent.	[133a, 151]
30	Lys acetylation	Fmoc‐Lys(Ac)‐OH	N	Accessibility of enantiomeric reagent.	[133b]
31	Asn *N*‐Glycosylation	Fmoc‐Asn(Glycan)−OH or Boc‐Asn(Xan)−OH (and) further glycosylation on‐resin or in solution.	N	Accessibility of enantiomeric reagent (would also require L‐sugars).	[53, 157]
32		Oligosaccharide coupled directly to free Asn side chain during Boc‐SPPS.	N	Accessibility of enantiomeric reagent (would also require L‐sugars).	[158]
33	Thr *O*‐Glycosylation	Fmoc‐Thr(Glycan)−OH	N	Accessibility of enantiomeric reagent (would also require L‐sugars).	[131b]
34	Cys *S*‐palmitoylation	Fmoc‐Cys(Mmt)−OH Mmt removal on‐resin with 2 % TFA Reaction with palmitic anhydride	N	Incompatible with NCL. Potentially viable for D‐protein synthesis via STL or Sec NCL.	[137a]
35		Fmoc‐Cys(palmityl)‐OH	N	Incompatible with NCL. Fmoc‐D‐Cys(palmityl) must be synthesized.	[137b]
36	Tyr sulfation	Fmoc‐Tyr(OTBS)−OH Deprotection and sulfation on‐resin with:	N	Accessibility of enantiomeric reagent.	[131b]
		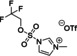			
		+DIEPA			
37	Ser phosphorylation	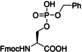	Y	Accessibility of enantiomeric reagent.	[18, 130b, 159]
38	Tyrosine phosphorylation	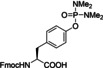	N	Accessibility of enantiomeric reagent.	[131a]

#### 3.2.1 Thioester preparation

Activated thioesters cannot be anchored at the C‐terminus during Fmoc‐SPPS due to their sensitivity to base treatment which is routinely used during deprotection. Consequently, inert precursors including the refined Dawson linker have been developed (Table 3, Entry 6). Following SPPS, the base‐stable 3,4‐diaminobenzoic acid (Dbz) derivative is activated and subjected to thiolysis to yield the thioester for native chemical ligation. More recently, a second generation Dawson linker recruits an N‐methylated amino group minimizing unwanted acylation when an excess of glycine reagent is used (Table 3, Entry 7).^[98]^ Assembly of peptides directly onto the linker amine is a simple and useful feature of the Dawson linker. Alternatively, the C‐terminal peptide hydrazide can be used to create a C‐terminal thioester (Table 3, Entry 5).^[99]^ Activated by the addition of sodium nitrite, the hydrazide can be oxidized into an acyl azide which can be subjected to thiolysis *in situ*.^[100]^ Hydrazine resins are prepared fresh before use, but it has recently been shown that resins can be prepared with Fmoc‐hydrazine facilitating long term storage and facile loading quantification.^[101]^

In the convergence of multiple peptide fragments to prepare larger synthetic proteins, some central fragments will be ligated at both N‐ and C‐termini. To prevent intramolecular side‐reactions (cyclization), the peptide can be prepared as a thioester surrogate, whereby a C‐terminal functional group remains inert in NCL and can be subsequently activated for the next ligation step. The peptide hydrazide is the most common choice in this instance due to the facile *in situ* activation and has been used to prepare numerous D‐proteins, including the mirror‐image TIGIT domain discussed earlier.^[77b]^ Other methods for generating thioester surrogates, such as SEA chemistry (Table 3, Entry 8),^[102]^ could also be used but have yet to be applied to D‐protein synthesis.

#### 3.2.2 N‐terminal cysteine protection

In the pursuit of more complex protein targets, sequential NCL steps will require orthogonal N‐terminal cysteine protecting groups for middle segments (Table 3, Entries 9–13). A common and reliable choice is the protection of cysteine in a thiazolidine ring (Thz), due to the facile conversion into cysteine, compatibility with ligation conditions and its commercial availability in both enantiomeric forms.^[103]^ However, Thz was found to be unstable under the oxidation conditions required for peptide hydrazide oxidation.^[99]^ Numerous efforts have been directed to the development of other orthogonal, N‐terminal cysteine protecting groups to facilitate sequential peptide hydrazide ligations (Table 3, Entries 9–12). Preparation of their enantiomeric counterpart is theoretically simple. Indeed, TFA‐Thz, which is stable to hydrazide oxidation, was used in the preparation of enantiomeric polymerase D‐Dpo4^[9]^ and ubiquitin.^[41]^

#### 3.2.3 NCL beyond cysteine

**Figure 8:**
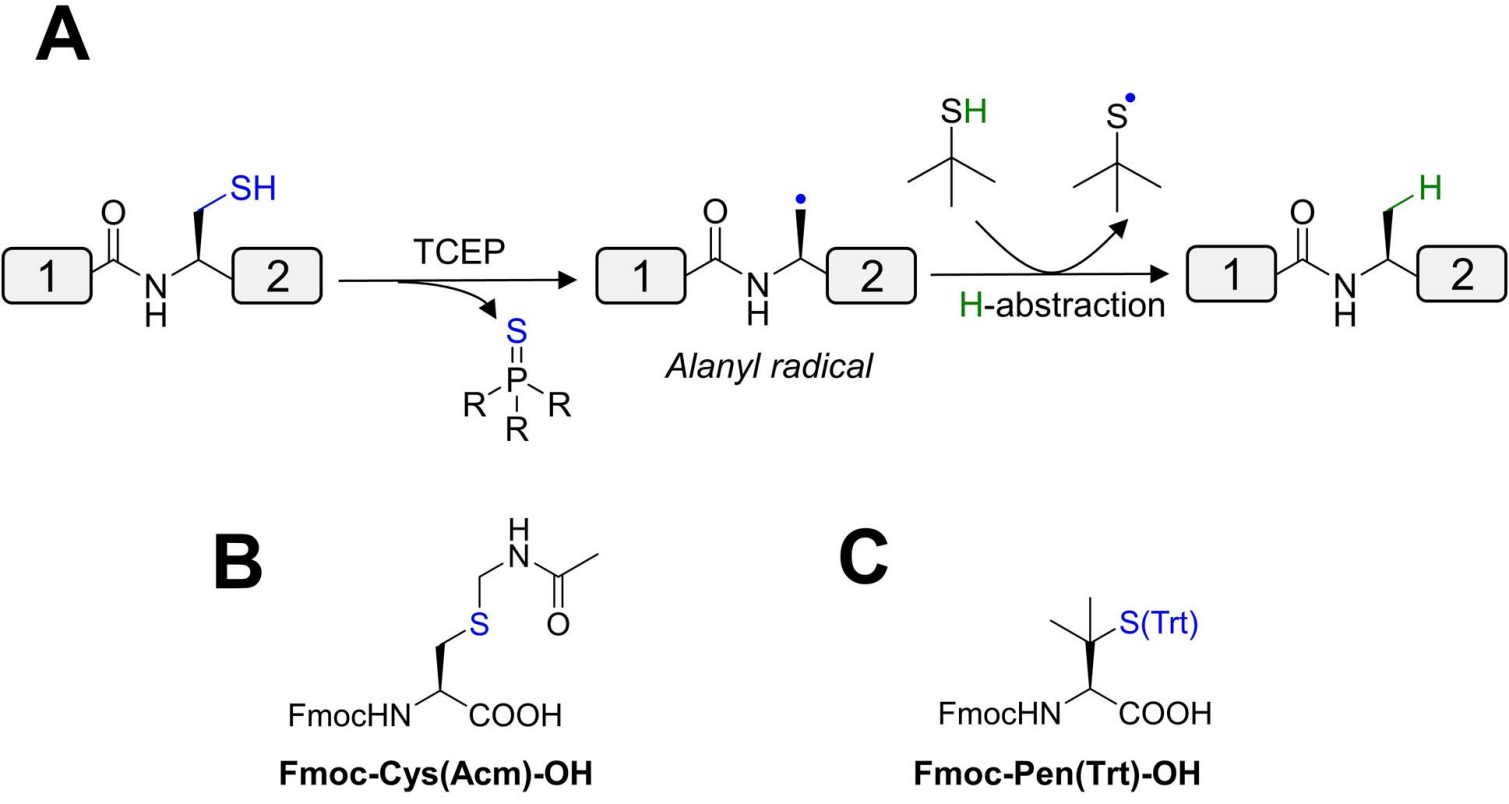
(A) Mechanism of metal‐free radical desulfurization, (B) acetamidomethyl cysteine (Cys(Acm)) and (C) penicillamine buildings blocks for Fmoc‐SPPS.

Due to the low abundance of cysteine residues in proteins (<2 %),^[104]^ significant efforts have been directed to link peptides with alternative amino acids.^[105]^ Desulfurization converts the reactive cysteine thiol CH_2_SH to the CH_3_ group of alanine,^[106]^ which is significantly higher in abundance (>8 %).^[107]^ Techniques include hydrogenation over Pd/Al_2_O_3_ or Raney nickel (Table 3, Entry 14).^[106]^ Given the issues associated with purity, a metal‐free desulfurization alternative has been developed through a free‐radical mechanism (Table 3, Entry 15; Figure 8A). However, native cysteines must be protected during desulfurization reactions. Acetamidomethyl cysteine (Cys(Acm)) is commonly employed in D‐protein synthesis, and is commercially available in both L‐ and D‐ enantiomers for Fmoc‐SPPS (Figure 8B).^[108]^ Following desulfurization, the Acm group can be removed using mercury acetate,^[137]^ iodine,^[138]^ or more recently, PdCl_2_.^[109]^

Thiols may also be inserted into other canonical amino acids and can be removed by desulfurization (for review, see ref. [110]). However, this approach has not been applied in D‐protein synthesis because of challenges associated with synthesizing the enantiomeric building blocks (Table 3, Entry 16). Commercially available D‐penicillamine (D‐Pen) may be a viable option for ligation at valine (Figure 8C),^[111]^ but this residue is associated with slow ligation kinetics with all C‐terminal thioester sites other than glycine.^[112]^ NCL can also proceed by employing a temporarily inserted thiol auxiliary which can be removed by acid cleavage.^[113]^ Such auxiliaries enable the generation of the lysine isopeptide bonds in the synthesis of branched ubiquitin chains (Table 3, Entry 28).[^28^, ^42^] Nevertheless, this approach has not yet been applied in the synthesis of the mirror‐image D‐counterparts (see section 3.3.2.3).^[42]^

#### 3.2.4 One‐pot approach

During the synthesis of large protein targets, it becomes advantageous to conduct steps in a ‘one‐pot’ fashion minimizing lengthy and yield‐reducing purification maneuvers. For example, directly after a NCL reaction, the desulfurization step can be performed in one pot, followed by Cys(Acm) deblocking without intermediate purification.^[109]^ A primary limitation of performing desulfurization immediately after NCL is that: a large excess of thiol catalyst such as 4‐mercaptophenylacetic acid (MPAA) is needed to improve ligation rates but they also quench the desulfurization reaction.^[114]^ Efforts have been directed toward finding new thiol catalysts that are compatible with desulfurization (Table 3, Entries 17–19). Methyl thioglycolate was found to not interfere with desulfurization whilst providing good catalytic properties, and it has been used in the synthesis of mirror‐image ubiquitin for racemic protein crystallography studies.^[41]^ Other efforts to reduce the number of HPLC purification steps include one‐pot ligations of numerous fragments and performing chemical ligations on a solid support.[^57^, ^115^]

#### 3.2.5 Enhancing solubility of hydrophobic peptide fragments

**Figure 9:**
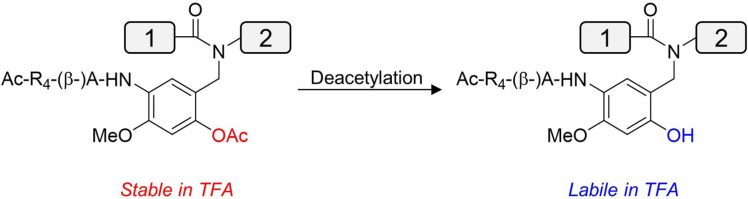
Second generation removable backbone modification. 4‐Methoxy‐5‐nitrosalicylaldehyde is installed onto backbone nitrogen during Fmoc‐SPPS, followed by assembly of peptide main chain and desired tag sequence. Reversible acetylation of phenol group controls TFA lability for tag removal.

The nature of protein has implications on the experimental design. Hydrophobic proteins such as membrane proteins can suffer from aggregation and poor solubility.^[116]^ A solution that can address these issues is to recruit the first‐generation removable backbone modification (RBM), which minimizes aggregation whilst allowing conjugation to a poly‐arginine solubility tag (Table 3, Entry 22).^[117]^ However, the RBM is installed via a removable glycine auxiliary and thus has limited scope. A second‐generation RBM was designed to be installed into all other amino acids, including the challenging Val‐Ile junction, making this highly attractive for the synthesis of membrane proteins (Table 3, Entry 23; Figure 9).^[118]^ The RBM tags have been employed in the synthesis of the mirror‐image TIGIT membrane protein domain.^[77b]^ In addition to RBMs, removable solubilizing tags could also be incorporated onto lysine side chains (Fmoc‐Ddae‐OH) or (Fmoc‐Ddap‐OH) using the ‘helping‐hand’ strategies (Table 3, Entries 20, 21).^[119]^ Whilst no D‐proteins are currently reported using this method, these tags could possibly be installed onto commercially available D‐amino acid building blocks. The lysine tags can also be employed to install click handles for templated chemical protein ligations.^[120]^

#### 3.2.6 Remarks

Since the advent of chemical ligation methods, protein synthesis has transformed into a rapidly evolving field of research. Particularly, native chemical ligation has facilitated the synthesis of numerous D‐proteins: enantiomeric venom toxins,[^30^, ^34^, ^35^, ^36^, ^38^, ^45^, ^48^, ^50^, ^52^, ^57^] growth factors,[^56^, ^72^, ^74^, ^75^] and enzymes,[^5^, ^7^, ^9^, ^12^, ^13^, ^14^, ^121^] including the 90 kDa split‐enzyme D‐*Pfu*.^[10]^ The pursuit of more complex D‐proteins is perplexed by their size and post‐translational modification status. Multi‐segment, one‐pot approaches can improve efficiency, but many recruit unique amino‐acid reagents that need to be prepared in the laboratories.^[122]^ Another issue involves the rates of ligation reactions, which can potentially be increased by adopting selenocysteine NCL,^[123]^ and template‐directed chemical ligations.^[124]^ Easier access to protected building blocks, and their commercial availability, will greatly improve the possibilities for D‐protein chemical synthesis. Design of synthetic routes to D‐proteins can also be assisted by computational approaches such as the open‐source ‘Alligator’ tool.^[125]^ Perhaps, the ultimate goal for D‐polypeptide production is to completely circumvent chemical synthesis, with the entire mirror‐image translational machinery (ribosome, rRNA, tRNA, tRNA synthetase, etc.) served as a replacement.

### 3.3 Transformation from D‐polypeptide to mirror‐image protein

A somewhat overlooked challenge is the complexity of protein folding that researchers may need to address during synthetic protein preparation.^[126]^ Typically, a solution of the protein in chaotropic conditions such as guanidine or urea is prepared and then diluted into a refolding buffer.^[127]^ Many larger proteins require chaperones for efficient folding, particularly *in vivo* where direct control of refolding conditions is limited. Recently, it was shown that the GroEL/ES chaperone protein can efficiently fold both enantiomers (L‐ and D‐) of synthetic DapA protein.^[121b]^ This observation suggested that the protein chaperone activity can be achiral and may find broad utility in the pursuit of mirror‐image life systems. Two specific challenges associated with protein folding that will be discussed here include correct oxidation of disulfide bonds and installation of post‐translational modifications.

#### 3.3.1 Disulfide bond formation

**Figure 10:**
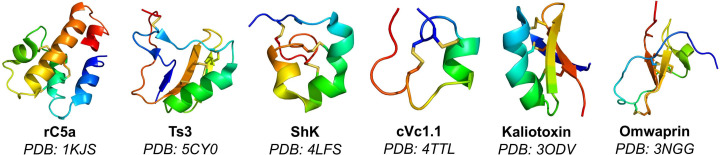
Examples of disulfide‐rich D‐proteins prepared by chemical synthesis, with structures resolved by racemic protein crystallography.

Correct folding of the disulfide bonds is case‐dependent, often requiring extensive screening and optimization for each protein. Nevertheless, preparation of enantiomeric disulfide‐rich proteins for racemic crystallography has broad utility in generating new structural insights (Table 1 and Figure 10).[^30^, ^34^, ^45^, ^50^, ^52^] One common method for disulfide bond formation involves diluting the reduced D‐polypeptide chain from chaotropic agents in the presence of reduced and oxidized thiols as redox reagents, as reported in the synthesis of D‐rC5a.^[35]^ However, misfolded D‐protein often arises as thermodynamically trapped by‐products containing mismatched disulfide bonds and adducts with thiol reagents.[^30^, ^45^] Oxidation by air or DMSO has been used, following careful optimization of buffer additives, reagent concentrations, and pH. However, this method often results in low yields (typically <50 %), requires large solvent volumes, and proceeds over several days.[^30^, ^45^, ^49^] To gain additional control, orthogonal cysteine protection followed by pairwise cysteine oxidation may be used. Recently, an orthogonal cysteine protection scheme was developed, encoding a rapid system for disulfide oxidation (Table 3, Entry 24). Using palladium and UV light mediated deprotections, it was shown that up to three correctly paired disulfide bonds could be formed in less than 13 minutes.^[128]^ The use of trityl (Trt) and acetamidomethyl (Acm) protecting groups has been reported for formation two disulfide bonds in a D‐protein (Table 3, Entry 24).^[52]^ Another protecting group that can be used is the 2‐nitrobenzyl group but must be chemically synthesized (Table 3, Entry 24).^[128]^ Disulfide bond formation can also be directed without the use of chaotropic agents or orthogonal protection schemes, such as cysteine‐penicillamine pairings or repeat‐proline (CPPC) motifs.^[129]^

#### 3.3.2 Post‐translational modifications

To achieve a true, mirror‐image protein with reciprocal stereospecific protein activity, PTMs must be incorporated into the D‐polypeptide product. Serine and tyrosine phosphorylation are common PTMs.^[130]^ Whilst the L‐serine equivalent (Table 3, Entry 37) is commercially available, the corresponding protected D‐Ser building block must be accessed through chemical synthesis.^[18]^ This has been demonstrated in the synthesis of mirror‐image ribosomal proteins which are essential in the binding of L‐RNA molecules.^[18]^ Phosphorylation and sulfation of tyrosine residues have been incorporated into chemical synthesis of L‐proteins^[131]^ using similar protected building blocks (Table 3, Entry 36 & 38). It is reasonable that equivalent building blocks can be prepared in D‐enantiomeric form. Indeed, tyrosine phosphorylation is a PTM observed in many transcriptional regulators and has been shown to impact DNA binding.^[130c]^

Glycosylation is another widespread PTM found in proteins.^[132]^ However, the sugars attached to the protein are chiral existing almost exclusively in D‐form.^[1a]^ Mirror‐image glycoprotein would require installation of L‐sugar polymers onto the D‐polypeptide and has not yet been reported. However, quasi‐racemic protein crystallography of synthetic glycoprotein Ser‐CCL1 could be facilitated using the un‐glycosylated D‐enantiomer.^[53]^ In many cases, glycans can play important roles in protein or nucleotide binding,^[132]^ and therefore achieving mirror‐image protein glycosylation is a key milestone in research surrounding D‐proteins, particularly in the area of MIPD.

**Figure 11:**
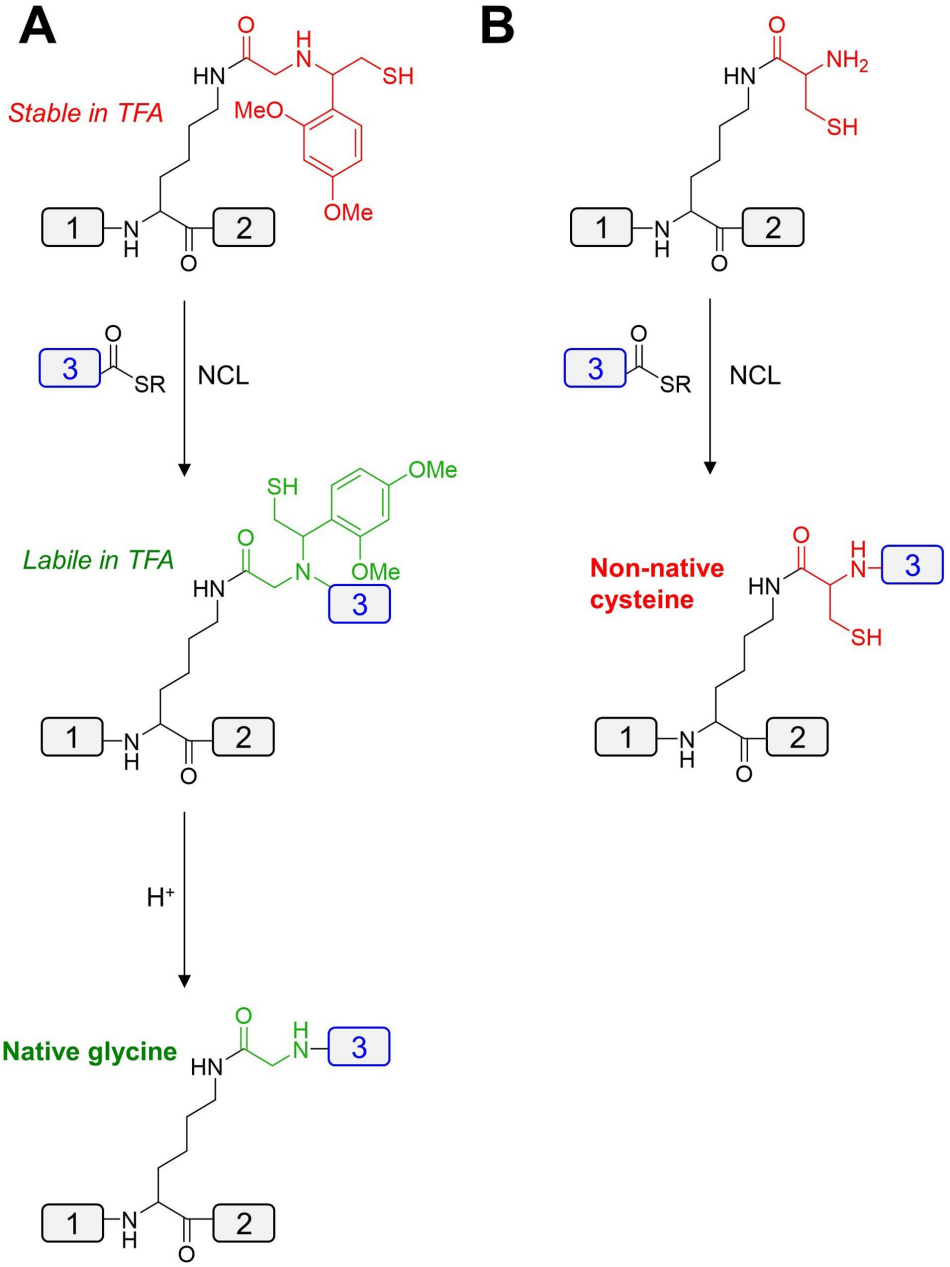
Methods for branched protein ligation used in preparation of poly‐ubiquitins; (A) glycyl auxiliary mediated NCL for preparation of branched L‐proteins and (B) cysteine mediated NCL for preparation of branched D‐proteins.

Other promising PTMs include lysine trimethylation or acetylation, both of which have been incorporated into the chemical synthesis of L‐histones (Table 3, Entries 29, 30).^[133]^ Preparation of the corresponding D‐histones would require the synthesis of the lysine building blocks in D‐form. A more challenging lysine PTM is site‐specific ubiquitination.^[134]^ Chemical synthesis of branched ubiquitin chains in native L‐form has been reported,^[135]^ typically employing a δ‐mercapto lysine to facilitate ligation, followed by desulfurization (Table 3, Entry 26).[^134^, ^136^] However, preparation of the branched ubiquitin chains for racemic protein crystallography utilized an auxiliary for NCL, following subsequent removal with TFA to generate a native glycine at the branched ligation site (Figure 11A).[^28^, ^42^] During preparation of the D‐enantiomers of ubiquitinated proteins, the auxiliary was replaced with a cysteine residue to circumvent the low efficiency of the ligations, leaving a non‐native cysteine as a scar at the ligation site (Figure 11B).^[42]^

Palmitoylation also serves as a promising PTM for incorporation into D‐proteins and has been applied to the synthesis of cysteine palmitoylated L‐proteins (Table 3, Entries 34, 35).^[137]^ Palmitic acid is achiral and could be conjugated to D‐cysteine or other D‐amino acid residues. Synthesis of palmitoylated D‐proteins may find use in racemic protein crystallography^[26]^ which has been applied to reveal the structure of transmembrane protein domains in racemic detergents.^[27]^ In addition, palmitoylation is a useful modification for pharmacokinetic optimization of peptide drugs,^[138]^ including the FDA‐approved Liraglutide.^[139]^ This modification may find use in prolonging the half‐life of D‐peptide drug candidates, as encountered with the conjugation of cholesterol to the promising D‐peptide drug candidate, CPT31.^[79]^

#### 3.3.3 Remarks

Folding of D‐polypeptide chains into the desired protein conformation is a challenging task, and often requires optimization in a case dependent manner. Protein refolding protocols can be pre‐established using the native recombinant L‐counterparts,^[140]^ and they are often sufficient to fold the corresponding D‐enantiomer. In the case where disulfide bond formation is involved, oxidants such as cystine or glutathione disulfide can be used.^[52]^ Alternatively, orthogonal protection schemes can be implemented.[^52^, ^128^] Most PTM installations can be achieved, particularly the achiral components such as phosphorylation.^[18]^ However, chiral PTMs such as glycosylation largely remains an unresolved synthetic challenge.^[53]^ A plausible solution would be to obtain a mirror‐image enzyme (such as *endo‐*glycoside hydrolase),^[141]^ capable of assembling the necessary polysaccharide building block from L‐sugars, much like the enantiomeric polymerase enzymes discussed earlier.[^9^, ^10^, ^13^, ^14^]

## 4 Perspectives

Applications of synthetic D‐protein enantiomers are vast but remains to be challenged by the difficulty in their preparation, particularly when their complexity increases with their size, folding and post‐translational modifications. The ability to translate L‐mRNA into D‐proteins will be truly revolutionary. The first milestone will be the complete assembly of an enantiomeric ribosome, comprised of D‐proteins and L‐rRNAs. Since three out of ∼50 mirror‐image *E. coli* ribosomal proteins^[18]^ and efficient L‐nucleotide polymerases[^7^, ^9^, ^10^, ^12^, ^13^, ^14^, ^16^] have been reported, it is anticipated that this ambition will be achieved in the future. Mirror‐image translation could then be achieved *in vitro*,^[142]^ using D‐aminoacyl‐tRNA synthetases to load the L‐tRNAs along with the mirror‐imaged translational factors. Subsequently, all D‐proteins needed in mirror‐image life, racemic protein crystallography and D‐targets for mirror‐image phage display may be obtained by suppling the exogenous L‐nucleic acids encoding the desired protein. Perhaps, preparation of entire D‐polypeptides can also be achieved using “Flexizyme” technology,^[143]^ which has been reported to incorporate D‐amino acids into peptide chains without using enantiomeric translation components.[^2b^, ^67a^, ^67b^] Chemical synthesis remains superior at atomistic control allowing researchers to incorporate building blocks without constraints associated with ribosome‐based systems. Complex D‐protein targets can be achieved via: engineering of split enzymes,^[10]^ mutational installation of suitable ligation sites^[10]^ and *in silico* design of accessible D‐enzymes.^[16]^ For protein crystallography, smaller D‐proteins can be used to facilitate crystallization of complex L‐proteins by quasi‐racemic protein crystallography.^[28]^ In addition, discovery of D‐peptide binders could be achieved via computational‐based approaches.[^88^, ^89^, ^144^] Together, while there are unsolved challenges, D‐polypeptide research remains to have strong potentials that can generate explosive impacts on numerous research topics.

## Conflict of interest

The authors declare no conflict of interest.

5

## Biographical Information

*Alexander Lander (left) is a PhD student at Cardiff University working under the supervision of Louis Luk. He obtained his BSc in Chemistry and MSc in Medicinal Chemistry from Cardiff University, with a research placement at Instituto Químico de Sarrià. His research focuses on utilizing D‐proteins for structural studies of antimicrobial proteins and development of D‐peptide inhibitors of new drug targets. Yi Jin (center) is a Wellcome Trust Sir Henry Dale Fellow at Manchester Institute of Biotechnology. She obtained her BSc and MSc in organic chemistry at Xiamen University, China, and her PhD in enzymology in the University of Sheffield under Jon P. Waltho and G. Mike Blackburn. After three years in protein crystallography and chemical biology with Gideon J. Davies, FRS, FMedSci, at the University of York, she moved to Cardiff University in 2017 as a tenured University Research Fellow and then in 2021 progressed to the University of Manchester. Her research focuses on investigating proteins that can provide solutions to antibiotic resistance via chemical biology that integrates chemical synthesis, protein crystallography and NMR spectroscopy, and molecular biology. Louis Y. P. Luk (right) is a senior lecturer at the School of Chemistry of Cardiff University. He obtained his BSc in Chemistry and Microbiology & Immunology at the University of British Columbia. He obtained his PhD at the same university under the supervisor of Martin E. Tanner, followed by post‐doctoral studies in the laboratories of Stephen B. H. Kent at the University of Chicago and Rudolf K. Allemann at Cardiff University. He became a University Research Fellow in 2015 and recently became Senior Lecturer at Cardiff University (2020). His current research combines his training, focusing on enzyme engineering, bioconjugation chemistry and protein synthesis*.

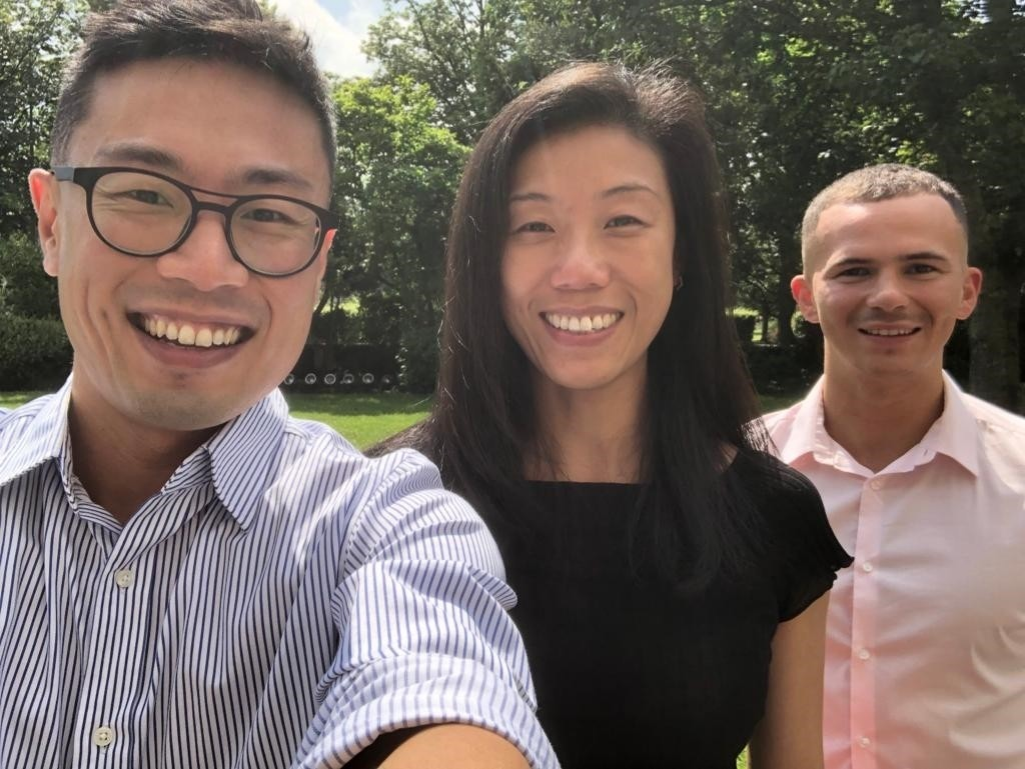

## Data Availability

Data sharing is not applicable to this article as no new data were created or analyzed in this study.

## References

[1a] V. A. Davankov , Symmetry-Basel 2018, 10 749;

[1b] G. Carenzi , S. Sacchi , M. Abbondi , L. Pollegioni , Amino Acids 2020, 52, 849–862. 32671478 10.1007/s00726-020-02873-w

[2a] L. M. Dedkova , N. E. Fahmi , S. Y. Golovine , S. M. Hecht , Biochemistry 2006, 45, 15541–15551; 17176075 10.1021/bi060986a

[2b] Y. Goto , H. Murakami , H. Suga , RNA 2008, 14, 1390–1398; 18515548 10.1261/rna.1020708 PMC2441986

[2c] S. K. Kuncha , S. P. Kruparani , R. Sankaranarayanan , J. Biol. Chem. 2019, 294, 16535–16548. 31591268 10.1074/jbc.REV119.008166 PMC6851308

[3] J. Kobayashi , Y. Shimizu , Y. Mutaguchi , K. Doi , T. Ohshima , J. Mol. Catal. B 2013, 94, 15–22.

[4] Y. Ogasawara , T. Dairi , Front. Microbiol. 2018, 9, 156. 29467749 10.3389/fmicb.2018.00156 PMC5808125

[5] R. Milton , S. Milton , S. Kent , Science 1992, 256, 1445–1448. 1604320 10.1126/science.1604320

[6] L. Pasteur, *Société Chimique de Paris* **1860**, *Reprint No. 14 (Alembic Club, 1905)*.

[7] M. Peplow , Nat. News 2016, 533, 303. 10.1038/nature.2016.19918 27193657

[8a] J. W. Szostak , D. P. Bartel , P. L. Luisi , Nature 2001, 409, 387–390; 11201752 10.1038/35053176

[8b] A. C. Forster , G. M. Church , Mol. Syst. Biol. 2006, 2, 45; 16924266 10.1038/msb4100090 PMC1681520

[8c] D. G. Gibson , J. I. Glass , C. Lartigue , V. N. Noskov , R.-Y. Chuang , M. A. Algire , G. A. Benders , M. G. Montague , L. Ma , M. M. Moodie , Science 2010, 329, 52–56. 20488990 10.1126/science.1190719

[9] W. Jiang , B. Zhang , C. Fan , M. Wang , J. Wang , Q. Deng , X. Liu , J. Chen , J. Zheng , L. Liu , T. F. Zhu , Cell Discov. 2017, 3, 17037. 29051832 10.1038/celldisc.2017.37 PMC5643884

[10] C. Fan , Q. Deng , T. F. Zhu , Nat. Biotechnol. 2021, 1548–1555. 34326549 10.1038/s41587-021-00969-6

[11] L. Schrödinger, W. DeLano, The PyMOL Molecular Graphics System, Version 2.0, **2015**.

[12] Z. Wang , W. Xu , L. Liu , T. F. Zhu , Nat. Chem. 2016, 8, 698–704. 27325097 10.1038/nchem.2517

[13] A. Pech , J. Achenbach , M. Jahnz , S. Schülzchen , F. Jarosch , F. Bordusa , S. Klussmann , Nucleic Acids Res. 2017, 45, 3997–4005. 28158820 10.1093/nar/gkx079 PMC5605242

[14] M. Wang , W. Jiang , X. Liu , J. Wang , B. Zhang , C. Fan , L. Liu , G. Pena-Alcantara , J.-J. Ling , J. Chen , T. F. Zhu , Chem 2019, 5, 848–857.

[15] J. Cline , J. C. Braman , H. H. Hogrefe , Nucleic Acids Res. 1996, 24, 3546–3551. 8836181 10.1093/nar/24.18.3546 PMC146123

[16] J. Weidmann , M. Schnölzer , P. E. Dawson , J. D. Hoheisel , Cell Chem. Biol. 2019, 26, 645–651.e643. 30880154 10.1016/j.chembiol.2019.02.008

[17] J. de la Cruz , K. Karbstein , J. L. Woolford Jr , Annu. Rev. Biochem. 2015, 84, 93–129. 25706898 10.1146/annurev-biochem-060614-033917 PMC4772166

[18] J.-J. Ling , C. Fan , H. Qin , M. Wang , J. Chen , P. Wittung-Stafshede , T. F. Zhu , Angew. Chem. Int. Ed. 2020, 59, 3724–3731; 10.1002/anie.201914799 PMC7217020 31841243

[19] S. Klinge , J. L. Woolford , Nat. Rev. Mol. Cell Biol. 2019, 20, 116–131. 30467428 10.1038/s41580-018-0078-y PMC7725133

[20] Y. Liu , E. Holmstrom , J. Zhang , P. Yu , J. Wang , M. A. Dyba , D. Chen , J. Ying , S. Lockett , D. J. Nesbitt , Nature 2015, 522, 368–372. 25938715 10.1038/nature14352 PMC4800989

[21] Y. Ishihama , T. Schmidt , J. Rappsilber , M. Mann , F. U. Hartl , M. J. Kerner , D. Frishman , BMC Genomics 2008, 9, 102. 18304323 10.1186/1471-2164-9-102 PMC2292177

[22] F. Rohden , J. D. Hoheisel , H.-J. Wieden , Trends Biochem. Sci. 2021, 46, 931–943. 34294544 10.1016/j.tibs.2021.06.006

[23] W.-S. Song , S.-X. Liu , C.-C. Chang , J. Org. Chem. 2018, 83, 14923–14932. 30474372 10.1021/acs.joc.8b02002

[24] T. O. Yeates , S. B. H. Kent , Annu. Rev. Biophys. 2012, 41, 41–61. 22443988 10.1146/annurev-biophys-050511-102333

[25] S. W. Wukovitz , T. O. Yeates , Nat. Struct. Biol. 1995, 2, 1062–1067. 8846217 10.1038/nsb1295-1062

[26] L. E. Zawadzke , J. M. Berg , Proteins Struct. Funct. Genet. 1993, 16, 301–305. 8346193 10.1002/prot.340160308

[27a] D. F. Kreitler , Z. Yao , J. D. Steinkruger , D. E. Mortenson , L. Huang , R. Mittal , B. R. Travis , K. T. Forest , S. H. Gellman , J. Am. Chem. Soc. 2019, 141, 1583–1592; 30645104 10.1021/jacs.8b11246 PMC6500429

[27b] D. E. Mortenson , J. D. Steinkruger , D. F. Kreitler , D. V. Perroni , G. P. Sorenson , L. Huang , R. Mittal , H. G. Yun , B. R. Travis , M. K. Mahanthappa , K. T. Forest , S. H. Gellman , Proc. Natl. Acad. Sci. USA 2015, 112, 13144–13149. 26460035 10.1073/pnas.1507918112 PMC4629355

[28] S. Gao , M. Pan , Y. Zheng , Y. C. Huang , Q. Y. Zheng , D. M. Sun , L. N. Lu , X. D. Tan , X. L. Tan , H. Lan , J. X. Wang , T. Wang , J. W. Wang , L. Liu , J. Am. Chem. Soc. 2016, 138, 14497–14502. 27768314 10.1021/jacs.6b09545

[29a] K. Mandal , B. L. Pentelute , D. Bang , Z. P. Gates , V. Y. Torbeev , S. B. H. Kent , Angew. Chem. Int. Ed. 2012, 51, 1481–1486; 10.1002/anie.201107846 22213444

[29b] B. L. Pentelute , Z. P. Gates , V. Tereshko , J. L. Dashnau , J. M. Vanderkooi , A. A. Kossiakoff , S. B. H. Kent , J. Am. Chem. Soc. 2008, 130, 9695–9701. 18598029 10.1021/ja8013538 PMC2719301

[30] J. R. Banigan , K. Mandal , M. R. Sawaya , V. Thammavongsa , A. P. A. Hendrickx , O. Schneewind , T. O. Yeates , S. B. H. Kent , Protein Sci. 2010, 19, 1840–1849. 20669184 10.1002/pro.468 PMC2998720

[31] A. J. Lander , X. Li , Y. Jin , L. Y. P. Luk , ChemRxiv preprint 2020, 10.26434/chemrxiv.12444554.v1.

[32] Y.-H. Huang , Q. Du , Z. Jiang , G. J. King , B. M. Collins , C. K. Wang , D. J. Craik , Molecules 2021, 26, 5554. 34577034 10.3390/molecules26185554 PMC8467136

[33] Q. Qu , S. Gao , F. Wu , M.-G. Zhang , Y. Li , L.-H. Zhang , D. Bierer , C.-L. Tian , J.-S. Zheng , L. Liu , Angew. Chem. Int. Ed. 2020, 59, 6037–6045; 10.1002/anie.201915358 32060988

[34] C. Zuo , B. Zhang , M. Wu , D. Bierer , J. Shi , G.-M. Fang , Chin. Chem. Lett. 2019, 31, 693–696.

[35] C. Zuo , W.-W. Shi , X.-X. Chen , M. Glatz , B. Riedl , I. Flamme , E. Pook , J. Wang , G.-M. Fang , D. Bierer , L. Liu , Sci. China Chem. 2019, 62, 1371–1378.

[36] K. W. Kurgan , A. F. Kleman , C. A. Bingman , D. F. Kreitler , B. Weisblum , K. T. Forest , S. H. Gellman , J. Am. Chem. Soc. 2019, 141, 7704–7708. 31059253 10.1021/jacs.9b02691 PMC6520119

[37] S. D. Ramalho , C. K. Wang , G. J. King , K. A. Byriel , Y.-H. Huang , V. S. Bolzani , D. J. Craik , J. Nat. Prod. 2018, 81, 2436–2445. 30345754 10.1021/acs.jnatprod.8b00447

[38] Q. Qu , S. Gao , Y.-M. Li , J. Pept. Sci. 2018, 24, e3112. 30009430 10.1002/psc.3112

[39] C. K. Wang , G. J. King , A. C. Conibear , M. C. Ramos , S. Chaousis , S. T. Henriques , D. J. Craik , J. Am. Chem. Soc. 2016, 138, 5706–5713. 27064294 10.1021/jacs.6b02575

[40] H. Yeung , C. J. Squire , Y. Yosaatmadja , S. Panjikar , G. López , A. Molina , E. N. Baker , P. W. R. Harris , M. A. Brimble , Angew. Chem. Int. Ed. 2016, 55, 7930–7933; 10.1002/anie.201602719 27145301

[41] Y.-C. Huang , C.-C. Chen , S. Gao , Y.-H. Wang , H. Xiao , F. Wang , C.-L. Tian , Y.-M. Li , Chem. Eur. J. 2016, 22, 7623–7628. 27075969 10.1002/chem.201600101

[42] M. Pan , S. Gao , Y. Zheng , X. Tan , H. Lan , X. Tan , D. Sun , L. Lu , T. Wang , Q. Zheng , Y. Huang , J. Wang , L. Liu , J. Am. Chem. Soc. 2016, 138, 7429–7435. 27268299 10.1021/jacs.6b04031

[43] D. E. Mortenson , K. A. Satyshur , I. A. Guzei , K. T. Forest , S. H. Gellman , J. Am. Chem. Soc. 2012, 134, 2473–2476. 22280019 10.1021/ja210045s PMC3351109

[44] D. F. Kreitler , D. E. Mortenson , K. T. Forest , S. H. Gellman , J. Am. Chem. Soc. 2016, 138, 6498–6505. 27171550 10.1021/jacs.6b01454 PMC5107306

[45] B. Dang , T. Kubota , K. Mandal , A. M. Correa , F. Bezanilla , S. B. Kent , Angew. Chem. Int. Ed. 2016, 55, 8639–8642; 10.1002/anie.201603420 PMC5001624 27244051

[46] Z. Hayouka , D. E. Mortenson , D. F. Kreitler , B. Weisblum , K. T. Forest , S. H. Gellman , J. Am. Chem. Soc. 2013, 135, 15738–15741. 24102563 10.1021/ja409082w PMC3928869

[47] Z. Hayouka , N. C. Thomas , D. E. Mortenson , K. A. Satyshur , B. Weisblum , K. T. Forest , S. H. Gellman , J. Am. Chem. Soc. 2015, 137, 11884–11887. 26369301 10.1021/jacs.5b07206 PMC4831726

[48] B. Dang , T. Kubota , K. Mandal , F. Bezanilla , S. B. Kent , J. Am. Chem. Soc. 2013, 135, 11911–11919. 23919482 10.1021/ja4046795 PMC3838204

[49] J. K. Murray , Y.-X. Qian , B. Liu , R. Elliott , J. Aral , C. Park , X. Zhang , M. Stenkilsson , K. Salyers , M. Rose , H. Li , S. Yu , K. L. Andrews , A. Colombero , J. Werner , K. Gaida , E. A. Sickmier , P. Miu , A. Itano , J. McGivern , C. V. Gegg , J. K. Sullivan , L. P. Miranda , J. Med. Chem. 2015, 58, 6784–6802. 26288216 10.1021/acs.jmedchem.5b00495

[50] B. Dang , R. Shen , T. Kubota , K. Mandal , F. Bezanilla , B. Roux , S. B. Kent , Angew. Chem. Int. Ed. 2017, 56, 3324–3328; 10.1002/anie.201612398 PMC5507063 28194851

[51] R. D. Bunker , K. Mandal , G. Bashiri , J. J. Chaston , B. L. Pentelute , J. S. Lott , S. B. H. Kent , E. N. Baker , Proc. Natl. Acad. Sci. USA 2015, 112, 4310–4315. 25831534 10.1073/pnas.1422387112 PMC4394262

[52] C. K. Wang , G. J. King , S. E. Northfield , P. G. Ojeda , D. J. Craik , Angew. Chem. Int. Ed. 2014, 53, 11236–11241; 10.1002/anie.201406563 25168664

[53] R. Okamoto , K. Mandal , M. R. Sawaya , Y. Kajihara , T. O. Yeates , S. B. H. Kent , Angew. Chem. Int. Ed. 2014, 53, 5194–5198; 10.1002/anie.201400679 24692304

[54] M. Avital-Shmilovici , K. Mandal , Z. P. Gates , N. B. Phillips , M. A. Weiss , S. B. H. Kent , J. Am. Chem. Soc. 2013, 135, 3173–3185. 23343390 10.1021/ja311408y PMC3625376

[55] K. Mandal , B. Dhayalan , M. Avital-Shmilovici , A. Tokmakoff , S. B. H. Kent , ChemBioChem 2016, 17, 421–425. 26707939 10.1002/cbic.201500600

[56] K. Mandal , M. Uppalapati , D. Ault-Riché , J. Kenney , J. Lowitz , S. S. Sidhu , S. B. H. Kent , Proc. Natl. Acad. Sci. USA 2012, 109, 14779–14784. 22927390 10.1073/pnas.1210483109 PMC3443191

[57] B. L. Pentelute , K. Mandal , Z. P. Gates , M. R. Sawaya , T. O. Yeates , S. B. H. Kent , Chem. Commun. 2010, 46, 8174–8176. 10.1039/c0cc03148h 20877851

[58] S. McNicholas , E. Potterton , K. S. Wilson , M. E. M. Noble , Acta Crystallogr. Sect. D 2011, 67, 386–394. 21460457 10.1107/S0907444911007281 PMC3069754

[59] N. Marín-Medina , D. A. Ramírez , S. Trier , C. Leidy , Appl. Microbiol. Biotechnol. 2016, 100, 10251–10263. 27837316 10.1007/s00253-016-7975-9

[60] G. L. Rosano , E. A. Ceccarelli , Front. Microbiol. 2014, 5, 172–172. 24860555 10.3389/fmicb.2014.00172 PMC4029002

[61] L. P. Kozlowski , Nucleic Acids Res. 2017, 45, D1112-D1116. 27789699 10.1093/nar/gkw978 PMC5210655

[62] S. Lien , H. B. Lowman , Trends Biotechnol. 2003, 21, 556–562. 14624865 10.1016/j.tibtech.2003.10.005

[63] J. L. Lau , M. K. Dunn , Bioorg. Med. Chem. 2018, 26, 2700–2707. 28720325 10.1016/j.bmc.2017.06.052

[64] M. Goodman , M. Chorev , Acc. Chem. Res. 1979, 12, 1–7.

[65a] C. Li , C. Y. Zhan , L. Zhao , X. S. Chen , W. Y. Lu , W. Y. Lu , Bioorg. Med. Chem. 2013, 21, 4045–4050; 23660015 10.1016/j.bmc.2013.04.039 PMC3726206

[65b] C. Li , M. Pazgier , J. Li , C. Q. Li , M. Liu , G. Z. Zou , Z. Y. Li , J. D. Chen , S. G. Tarasov , W. Y. Lu , W. Y. Lu , J. Biol. Chem. 2010, 285, 19572–19581. 20382735 10.1074/jbc.M110.116814 PMC2885236

[66] J. J. Miles , M. P. Tan , G. Dolton , E. S. J. Edwards , S. A. E. Galloway , B. Laugel , M. Clement , J. Makinde , K. Ladell , K. K. Matthews , T. S. Watkins , K. Tungatt , Y. Wong , H. S. Lee , R. J. Clark , J. M. Pentier , M. Attaf , A. Lissina , A. Ager , A. Gallimore , P. J. Rizkallah , S. Gras , J. Rossjohn , S. R. Burrows , D. K. Cole , D. A. Price , A. K. Sewell , J. Clin. Invest. 2018, 128, 1569–1580. 29528337 10.1172/JCI91512 PMC5873848

[67a] S. Imanishi , T. Katoh , Y. Yin , M. Yamada , M. Kawai , H. Suga , J. Am. Chem. Soc. 2021, 143, 5680–5684; 33822597 10.1021/jacs.1c02593

[67b] W. Liu , S. J. de Veer , Y.-H. Huang , T. Sengoku , C. Okada , K. Ogata , C. N. Zdenek , B. G. Fry , J. E. Swedberg , T. Passioura , D. J. Craik , H. Suga , J. Am. Chem. Soc. 2021, 143, 18481–18489; 34723512 10.1021/jacs.1c07574

[67c] T. Katoh , H. Suga , J. Am. Chem. Soc. 2022, 144, 2069–2072; 35099961 10.1021/jacs.1c12133

[67d] T. Katoh , H. Suga , Annu. Rev. Biochem. 2022, 91, 221–243. 35729073 10.1146/annurev-biochem-040320-103817

[68] G. Bhardwaj , V. K. Mulligan , C. D. Bahl , J. M. Gilmore , P. J. Harvey , O. Cheneval , G. W. Buchko , S. V. S. R. K. Pulavarti , Q. Kaas , A. Eletsky , P.-S. Huang , W. A. Johnsen , P. Greisen, Jr. , G. J. Rocklin , Y. Song , T. W. Linsky , A. Watkins , S. A. Rettie , X. Xu , L. P. Carter , R. Bonneau , J. M. Olson , E. Coutsias , C. E. Correnti , T. Szyperski , D. J. Craik , D. Baker , Nature 2016, 538, 329–335. 27626386 10.1038/nature19791 PMC5161715

[69] T. N. M. Schumacher , L. M. Mayr , D. L. Minor , M. A. Milhollen , M. W. Burgess , P. S. Kim , Science 1996, 271, 1854–1857. 8596952 10.1126/science.271.5257.1854

[70] E. Witsch , M. Sela , Y. Yarden , Physiology 2010, 25, 85–101. 20430953 10.1152/physiol.00045.2009 PMC3062054

[71] E. R. Verhaar , A. W. Woodham , H. L. Ploegh , Semin. Immunol. 2020, 101425. 33272897 10.1016/j.smim.2020.101425 PMC8164649

[72] C. Díaz-Perlas , M. Varese , S. Guardiola , M. Sánchez-Navarro , J. García , M. Teixidó , E. Giralt , ChemBioChem 2019, 20, 2079–2084. 31268623 10.1002/cbic.201900355

[73] M. Uppalapati , D. J. Lee , K. Mandal , H. Li , L. P. Miranda , J. Lowitz , J. Kenney , J. J. Adams , D. Ault-Riché , S. B. Kent , S. S. Sidhu , ACS Chem. Biol. 2016, 11, 1058–1065. 26745345 10.1021/acschembio.5b01006

[74] M. Uppalapati , D. J. Lee , K. Mandal , H. Li , L. P. Miranda , J. Lowitz , J. Kenney , J. J. Adams , D. Ault-Riché , S. B. H. Kent , S. S. Sidhu , ACS Chem. Biol. 2016, 11, 1058–1065. 26745345 10.1021/acschembio.5b01006

[75] P. S. Marinec , K. E. Landgraf , M. Uppalapati , G. Chen , D. Xie , Q. Jiang , Y. Zhao , A. Petriello , K. Deshayes , S. B. H. Kent , D. Ault-Riche , S. S. Sidhu , ACS Chem. Biol. 2021, 16, 548–556. 33621466 10.1021/acschembio.1c00017

[76] P. Sharma , J. P. Allison , Science 2015, 348, 56–61. 25838373 10.1126/science.aaa8172

[77a] H.-N. Chang , B.-Y. Liu , Y.-K. Qi , Y. Zhou , Y.-P. Chen , K.-M. Pan , W.-W. Li , X.-M. Zhou , W.-W. Ma , C.-Y. Fu , Y.-M. Qi , L. Liu , Y.-F. Gao , Angew. Chem. Int. Ed. 2015, 54, 11760–11764; 10.1002/anie.201506225 26259671

[77b] X. Zhou , C. Zuo , W. Li , W. Shi , X. Zhou , H. Wang , S. Chen , J. Du , G. Chen , W. Zhai , W. Zhao , Y. Wu , Y. Qi , L. Liu , Y. Gao , Angew. Chem. Int. Ed. 2020, 59, 15114–15118; 10.1002/anie.202002783 32386245

[78a] B. D. Welch , A. P. VanDemark , A. Heroux , C. P. Hill , M. S. Kay , Proc. Natl. Acad. Sci. USA 2007, 104, 16828–16833; 17942675 10.1073/pnas.0708109104 PMC2040420

[78b] B. D. Welch , J. N. Francis , J. S. Redman , S. Paul , M. T. Weinstock , J. D. Reeves , Y. S. Lie , F. G. Whitby , D. M. Eckert , C. P. Hill , M. J. Root , M. S. Kay , J. Virol. 2010, 84, 11235–11244. 20719956 10.1128/JVI.01339-10 PMC2953169

[79] J. S. Redman , J. N. Francis , R. Marquardt , D. Papac , A. L. Mueller , D. M. Eckert , B. D. Welch , M. S. Kay , Mol. Pharm. 2018, 15, 1169–1179. 29436835 10.1021/acs.molpharmaceut.7b01004 PMC5893306

[80a] C. Dammers , D. Yolcu , L. Kukuk , D. Willbold , M. Pickhardt , E. Mandelkow , A. H. C. Horn , H. Sticht , M. N. Malhis , N. Will , J. Schuster , S. A. Funke , PLoS One 2016, 11, 18; 10.1371/journal.pone.0167432 PMC5179029 28006031

[80b] X. C. Zhang , X. Y. Zhang , M. L. Zhong , P. Zhao , C. Guo , Y. Li , T. Wang , H. L. Gao , ACS Chem. Neurosci. 2020, 11, 4240–4253. 33284003 10.1021/acschemneuro.0c00518

[81] P. E. Kolkwitz , J. Mohrlüder , D. Willbold , Biomol. Eng. 2022, 12, 157. 10.3390/biom12020157 PMC8961585 35204656

[82] M. Nonaka , H. Mabashi-Asazuma , D. L. Jarvis , K. Yamasaki , T. O. Akama , M. Nagaoka , T. Sasai , I. Kimura-Takagi , Y. Suwa , T. Yaegashi , C.-T. Huang , C. Nishizawa-Harada , M. N. Fukuda , PLoS One 2021, 16, e0241157. 33406123 10.1371/journal.pone.0241157 PMC7787448

[83] M. Malhis , S. Kaniyappan , I. Aillaud , R. R. Chandupatla , L. M. Ramirez , M. Zweckstetter , A. H. C. Horn , E. Mandelkow , H. Sticht , S. A. Funke , ChemBioChem 2021, 22, 3049–3059. 34375027 10.1002/cbic.202100287 PMC8596876

[84] Z. Li , J. Xie , S. Peng , S. Liu , Y. Wang , W. Lu , J. Shen , C. Li , Bioconjugate Chem. 2017, 28, 2167–2179. 10.1021/acs.bioconjchem.7b00326 28715634

[85] S. Rudolph , A. N. Klein , M. Tusche , C. Schlosser , A. Elfgen , O. Brener , C. Teunissen , L. Gremer , S. A. Funke , J. Kutzsche , D. Willbold , PLoS One 2016, 11, e0147470. 26840229 10.1371/journal.pone.0147470 PMC4740492

[86] M. Liu , C. Li , M. Pazgier , C. Li , Y. Mao , Y. Lv , B. Gu , G. Wei , W. Yuan , C. Zhan , W. Y. Lu , W. Lu , Proc. Natl. Acad. Sci. USA 2010, 107, 14321–14326. 20660730 10.1073/pnas.1008930107 PMC2922601

[87] C. H. Wong , K. W. Siah , A. W. Lo , Biostatistics 2018, 20, 273–286. 10.1093/biostatistics/kxx069 PMC6409418 29394327

[88a] H. Engel , F. Guischard , F. Krause , J. Nandy , P. Kaas , N. Höfflin , M. Köhn , N. Kilb , K. Voigt , S. Wolf , T. Aslan , F. Baezner , S. Hahne , C. Ruckes , J. Weygant , A. Zinina , E. B. Akmeriç , E. B. Antwi , D. Dombrovskij , P. Franke , K. L. Lesch , N. Vesper , D. Weis , N. Gensch , B. Di Ventura , M. A. Öztürk , Synth. Syst. Biotechnol. 2021, 6, 402–413; 34901479 10.1016/j.synbio.2021.11.004 PMC8632724

[88b] M. Garton , S. Nim , T. A. Stone , K. E. Wang , C. M. Deber , P. M. Kim , Proc. Natl. Acad. Sci. USA 2018, 115, 1505–1510. 29378946 10.1073/pnas.1711837115 PMC5816147

[89] J. E. Hernández González , R. J. Eberle , D. Willbold , M. A. Coronado , Front. Mol. Biosci. 2022, 8, 816166. 35187076 10.3389/fmolb.2021.816166 PMC8852625

[90] M. Garton , M. Sayadi , P. M. Kim , PLoS One 2017, 12, e0187524. 29108013 10.1371/journal.pone.0187524 PMC5673230

[91] R. B. Merrifield , J. Am. Chem. Soc. 1963, 85, 2149–2154.

[92] R. Behrendt , P. White , J. Offer , J. Pept. Sci. 2016, 22, 4–27. 26785684 10.1002/psc.2836 PMC4745034

[93] E. Valeur , M. Bradley , Chem. Soc. Rev. 2009, 38, 606–631. 19169468 10.1039/b701677h

[94] A. P. Tofteng , L. Malik , S. L. Pedersen , K. K. Sorensen , K. J. Jensen , Chim. Oggi 2011, 29, 28–31.

[95] J. M. Collins , K. A. Porter , S. K. Singh , G. S. Vanier , Org. Lett. 2014, 16, 940–943. 24456219 10.1021/ol4036825

[96] M. D. Simon , P. L. Heider , A. Adamo , A. A. Vinogradov , S. K. Mong , X. Li , T. Berger , R. L. Policarpo , C. Zhang , Y. Zou , X. Liao , A. M. Spokoyny , K. F. Jensen , B. L. Pentelute , ChemBioChem 2014, 15, 713–720. 24616230 10.1002/cbic.201300796 PMC4045704

[97a] P. Dawson , T. Muir , I. Clark-Lewis , S. Kent , Science 1994, 266, 776–779; 7973629 10.1126/science.7973629

[97b] S. B. Kent , Chem. Soc. Rev. 2009, 38, 338–351. 19169452 10.1039/b700141j

[98] J. B. Blanco-Canosa , B. Nardone , F. Albericio , P. E. Dawson , J. Am. Chem. Soc. 2015, 137, 7197–7209. 25978693 10.1021/jacs.5b03504

[99] G.-M. Fang , Y.-M. Li , F. Shen , Y.-C. Huang , J.-B. Li , Y. Lin , H.-K. Cui , L. Liu , Angew. Chem. Int. Ed. 2011, 50, 7645–7649; 10.1002/anie.201100996 21648030

[100] J.-S. Zheng , S. Tang , Y.-K. Qi , Z.-P. Wang , L. Liu , Nat. Protoc. 2013, 8, 2483–2495. 24232250 10.1038/nprot.2013.152

[101] Y.-C. Huang , C.-C. Chen , S.-J. Li , S. Gao , J. Shi , Y.-M. Li , Tetrahedron 2014, 70, 2951–2955.

[102] O. Melnyk , C. Simonneau , J. Vicogne , in Chemical Ligation, Wiley-VCH, Weinheim, 2017, pp. 89–123.

[103] D. Bang , S. B. Kent , Angew. Chem. Int. Ed. 2004, 43, 2534–2538; 10.1002/anie.200353540 15127445

[104] J. M. Chalker , G. J. L. Bernardes , Y. A. Lin , B. G. Davis , Chem. Asian J. 2009, 4, 630–640. 19235822 10.1002/asia.200800427

[105] V. Agouridas , O. El Mahdi , V. Diemer , M. Cargoët , J.-C. M. Monbaliu , O. Melnyk , Chem. Rev. 2019, 119, 7328–7443. 31050890 10.1021/acs.chemrev.8b00712

[106] L. Z. Yan , P. E. Dawson , J. Am. Chem. Soc. 2001, 123, 526–533. 11456564 10.1021/ja003265m

[107] R. F. Doolittle , in Prediction of Protein Structure and the Principles of Protein Conformation (Ed.: G. D. Fasman ), Springer US, 1989, pp. 599–623.

[108] B. L. Pentelute , S. B. H. Kent , Org. Lett. 2007, 9, 687–690. 17286375 10.1021/ol0630144

[109] S. K. Maity , M. Jbara , S. Laps , A. Brik , Angew. Chem. Int. Ed. 2016, 55, 8108–8112; 10.1002/anie.201603169 27126503

[110] K. Jin , X. Li , Chem. Eur. J. 2018, 24, 17397–17404. 29947435 10.1002/chem.201802067

[111] C. Haase , H. Rohde , O. Seitz , Angew. Chem. Int. Ed. 2008, 47, 6807–6810; 10.1002/anie.200801590 18626881

[112] J. Chen , Q. Wan , Y. Yuan , J. Zhu , S. J. Danishefsky , Angew. Chem. Int. Ed. 2008, 47, 8521–8524; 10.1002/anie.200803523 PMC2756581 18833563

[113] S. S. Kulkarni , J. Sayers , B. Premdjee , R. J. Payne , Nat. Chem. Rev. 2018, 2, 0122.

[114] E. C. B. Johnson , S. B. H. Kent , J. Am. Chem. Soc. 2006, 128, 6640–6646. 16704265 10.1021/ja058344i

[115] M. Jbara , M. Seenaiah , A. Brik , Chem. Commun. 2014, 50, 12534–12537. 10.1039/c4cc06499b 25196573

[116] D. Olschewski , C. F. Becker , Mol. BioSyst. 2008, 4, 733–740. 18563247 10.1039/b803248c

[117] J.-S. Zheng , M. Yu , Y.-K. Qi , S. Tang , F. Shen , Z.-P. Wang , L. Xiao , L. Zhang , C.-L. Tian , L. Liu , J. Am. Chem. Soc. 2014, 136, 3695–3704. 24559202 10.1021/ja500222u

[118] J.-S. Zheng , Y. He , C. Zuo , X.-Y. Cai , S. Tang , Z. A. Wang , L.-H. Zhang , C.-L. Tian , L. Liu , J. Am. Chem. Soc. 2016, 138, 3553–3561. 26943264 10.1021/jacs.6b00515

[119a] M. M. Disotuar , M. E. Petersen , J. M. Nogueira , M. S. Kay , D. H. Chou , Org. Biomol. Chem. 2019, 17, 1703–1708; 29947407 10.1039/c8ob01212a PMC6310105

[119b] J. M. Fulcher , M. E. Petersen , R. J. Giesler , Z. S. Cruz , D. M. Eckert , J. N. Francis , E. M. Kawamoto , M. T. Jacobsen , M. S. Kay , Org. Biomol. Chem. 2019, 17, 10237–10244. 31793605 10.1039/c9ob02012h

[120] P. W. Erickson , J. M. Fulcher , P. Spaltenstein , M. S. Kay , Bioconjugate Chem. 2021, 32, 2233–2244. 10.1021/acs.bioconjchem.1c00403 PMC9769386 34619957

[121a] J. Weidmann , M. Schnölzer , P. E. Dawson , J. D. Hoheisel , Cell Chem. Biol. 2019, 26, 645–651.e643; 30880154 10.1016/j.chembiol.2019.02.008

[121b] M. T. Weinstock , M. T. Jacobsen , M. S. Kay , Proc. Natl. Acad. Sci. USA 2014, 111, 11679–11684. 25071217 10.1073/pnas.1410900111 PMC4136631

[122] L. Raibaut , N. Ollivier , O. Melnyk , Chem. Soc. Rev. 2012, 41, 7001–7015. 22935750 10.1039/c2cs35147a

[123a] N. Metanis , E. Keinan , P. E. Dawson , Angew. Chem. Int. Ed. 2010, 49, 7049–7053; 10.1002/anie.201001900 PMC4459706 20715234

[123b] O. Firstova , V. Agouridas , V. Diemer , O. Melnyk , in Chemical Protein Synthesis (Ed.: X. Li ), Springer US, 2022, pp. 213–239; 10.1007/978-1-0716-2489-0_15 35761052

[123c] M. Cargoët , V. Diemer , L. Raibaut , E. Lissy , B. Snella , V. Agouridas , O. Melnyk , in Peptide and Protein Engineering: From Concepts to Biotechnological Applications (Eds.: O. Iranzo , A. C. Roque ), Springer US, 2020, pp. 1–12;

[123d] V. Diemer , N. Ollivier , B. Leclercq , H. Drobecq , J. Vicogne , V. Agouridas , O. Melnyk , Nat. Commun. 2020, 11, 2558. 32444769 10.1038/s41467-020-16359-6 PMC7244499

[124] O. Vázquez , O. Seitz , J. Pept. Sci. 2014, 20, 78–86. 24395765 10.1002/psc.2602

[125] M. T. Jacobsen , P. W. Erickson , M. S. Kay , Bioorg. Med. Chem. 2017, 25, 4946–4952. 28651912 10.1016/j.bmc.2017.05.061 PMC5860884

[126] C. L. Lee , H. Liu , C. T. T. Wong , H. Y. Chow , X. Li , J. Am. Chem. Soc. 2016, 138, 10477–10484. 27479006 10.1021/jacs.6b04238

[127a] M. Bacchi , M. Jullian , S. Sirigu , B. Fould , T. Huet , L. Bruyand , M. Antoine , L. Vuillard , L. Ronga , L. M. G. Chavas , O. Nosjean , G. Ferry , K. Puget , J. A. Boutin , Protein Sci. 2016, 25, 2225–2242; 27670942 10.1002/pro.3051 PMC5119562

[127b] H. P. Sørensen , H. U. Sperling-Petersen , K. K. Mortensen , Protein Expression Purif. 2003, 31, 149–154. 10.1016/s1046-5928(03)00133-5 12963352

[128] S. Laps , F. Atamleh , G. Kamnesky , H. Sun , A. Brik , Nat. Commun. 2021, 12, 870. 33558523 10.1038/s41467-021-21209-0 PMC7870662

[129a] S. Yao , A. Moyer , Y. Zheng , Y. Shen , X. Meng , C. Yuan , Y. Zhao , H. Yao , D. Baker , C. Wu , Nat. Commun. 2022, 13, 1539; 35318337 10.1038/s41467-022-29210-x PMC8941120

[129b] Y. Wu , S. Fan , M. Dong , J. Li , C. Kong , J. Zhuang , X. Meng , S. Lu , Y. Zhao , C. Wu , Chem. Sci. 2022, 13, 7780–7789. 35865895 10.1039/d2sc00924b PMC9258321

[130a] G. J. Pagano , R. J. Arsenault , Expert Rev. Proteomics 2019, 16, 431–441; 30920853 10.1080/14789450.2019.1601015

[130b] S. Bondalapati , W. Mansour , M. A. Nakasone , S. K. Maity , M. H. Glickman , A. Brik , Chem. Eur. J. 2015, 21, 7360–7364; 25829361 10.1002/chem.201500540

[130c] L. J. Getz , C. S. Runte , J. K. Rainey , N. A. Thomas , J. Bacteriol. 2019, 201, e00205–00219. 31262836 10.1128/JB.00205-19 PMC6755747

[131a] T. M. T. Jensen , C. R. O. Bartling , O. A. Karlsson , E. Åberg , L. M. Haugaard-Kedström , K. Strømgaard , P. Jemth , ACS Chem. Biol. 2021, 16, 1191–1200; 34161732 10.1021/acschembio.1c00176 PMC8291497

[131b] Y. S. Y. Hsieh , L. C. Wijeyewickrema , B. L. Wilkinson , R. N. Pike , R. J. Payne , Angew. Chem. Int. Ed. 2014, 53, 3947–3951; 10.1002/anie.201310777 24615823

[132] E. J. Kim , M. R. Bond , D. C. Love , J. A. Hanover , Crit. Rev. Biochem. Mol. Biol. 2014, 49, 327–342. 25039763 10.3109/10409238.2014.931338 PMC6396312

[133a] N. Guidotti , C. C. Lechner , A. L. Bachmann , B. Fierz , ChemBioChem 2019, 20, 1124–1128; 30615245 10.1002/cbic.201800744

[133b] J. C. Shimko , J. A. North , A. N. Bruns , M. G. Poirier , J. J. Ottesen , J. Mol. Biol. 2011, 408, 187–204. 21310161 10.1016/j.jmb.2011.01.003 PMC3815667

[134] W. J. Gui , G. A. Davidson , Z. H. Zhuang , RSC Chem. Biol. 2021, 2, 450–467. 34381999 10.1039/d0cb00215a PMC8323803

[135] Y. Zhou , Q. Xie , H. Wang , H. Sun , J. Pept. Sci. 2022, 28, e3367. 34514672 10.1002/psc.3367

[136a] K. Ajish Kumar , M. Haj-Yahya , D. Olschewski , H. A. Lashuel , A. Brik , Angew. Chem. 2009, 121, 8234–8238; 10.1002/anie.200902936 19780082

[136b] F. El Oualid , R. Merkx , R. Ekkebus , D. S. Hameed , J. J. Smit , A. de Jong , H. Hilkmann , T. K. Sixma , H. Ovaa , Angew. Chem. 2010, 122, 10347–10351; 10.1002/anie.201005995 PMC3021723 21117055

[136c] K. A. Kumar , S. N. Bavikar , L. Spasser , T. Moyal , S. Ohayon , A. Brik , Angew. Chem. 2011, 123, 6261–6265; 10.1002/anie.201101920 21591043

[136d] K. S. A. Kumar , L. Spasser , L. A. Erlich , S. N. Bavikar , A. Brik , Angew. Chem. Int. Ed. 2010, 49, 9126–9131; 10.1002/anie.201003763 20815002

[136e] M. P. C. Mulder , F. El Oualid , J. ter Beek , H. Ovaa , ChemBioChem 2014, 15, 946–949; 24623714 10.1002/cbic.201402012 PMC4159580

[136f] H. Sun , A. Brik , Acc. Chem. Res. 2019, 52, 3361–3371. 31536331 10.1021/acs.accounts.9b00372

[137a] D.-L. Huang , C. Montigny , Y. Zheng , V. Beswick , Y. Li , X.-X. Cao , T. Barbot , C. Jaxel , J. Liang , M. Xue , C.-L. Tian , N. Jamin , J.-S. Zheng , Angew. Chem. Int. Ed. 2020, 59, 5178–5184; 10.1002/anie.201914836 31846559

[137b] T. S. Chisholm , S. S. Kulkarni , K. R. Hossain , F. Cornelius , R. J. Clarke , R. J. Payne , J. Am. Chem. Soc. 2020, 142, 1090–1100. 31840988 10.1021/jacs.9b12558

[138] E. E. Mulvihill , Curr. Opin. Lipidol. 2018, 29, 95–103. 29432213 10.1097/MOL.0000000000000495 PMC5882252

[139] L. B. Knudsen , J. Lau , Front. Endocrinol. 2019, 10, 155. 10.3389/fendo.2019.00155 PMC6474072 31031702

[140] M. S. Hashemzadeh , M. Mohammadi , H. E. G. Ghaleh , M. Sharti , A. Choopani , A. K. Panda , Protein Pept. Lett. 2021, 28, 122–130. 32729411 10.2174/0929866527999200729182831

[141] S. Kobayashi , Proc. Jpn. Acad. Ser. B 2007, 83, 215–247. 24367148 10.2183/pjab/83.215 PMC3859292

[142] D. Garenne , M. C. Haines , E. F. Romantseva , P. Freemont , E. A. Strychalski , V. Noireaux , Nat. Rev. Methods Primers 2021, 1, 49.

[143] Y. Goto , T. Katoh , H. Suga , Nat. Protoc. 2011, 6, 779–790. 21637198 10.1038/nprot.2011.331

[144] D. N. Woolfson , J. Mol. Biol. 2021, 433, 167160. 34298061 10.1016/j.jmb.2021.167160

[145] H. Liu , X. Li , Acc. Chem. Res. 2018, 51, 1643–1655. 29979577 10.1021/acs.accounts.8b00151

[146] F. Rohrbacher , T. G. Wucherpfennig , J. W. Bode , Top. Curr. Chem. 2015, 363, 1–31. 25761549 10.1007/128_2014_597

[147a] J. B. Blanco-Canosa , P. E. Dawson , Angew. Chem. Int. Ed. 2008, 47, 6851–6855; 10.1002/anie.200705471 PMC3182823 18651678

[147b] T. Noguchi , H. Ishiba , K. Honda , Y. Kondoh , H. Osada , H. Ohno , N. Fujii , S. Oishi , Bioconjugate Chem. 2017, 28, 609–619. 10.1021/acs.bioconjchem.6b00692 28032751

[148] L. Raibaut , H. Adihou , R. Desmet , A. F. Delmas , V. Aucagne , O. Melnyk , Chem. Sci. 2013, 4, 4061–4066.

[149] S. Tang , Y.-Y. Si , Z.-P. Wang , K.-R. Mei , X. Chen , J.-Y. Cheng , J.-S. Zheng , L. Liu , Angew. Chem. Int. Ed. 2015, 54, 5713–5717; 10.1002/anie.201500051 25772600

[150] M. Pan , Y. He , M. Wen , F. Wu , D. Sun , S. Li , L. Zhang , Y. Li , C. Tian , Chem. Commun. 2014, 50, 5837–5839. 10.1039/c4cc00779d 24619065

[151] J. Li , Y. Li , Q. He , Y. Li , H. Li , L. Liu , Org. Biomol. Chem. 2014, 12, 5435–5441. 24934931 10.1039/c4ob00715h

[152] Q. Wan , S. J. Danishefsky , Angew. Chem. Int. Ed. 2007, 46, 9248–9252; 10.1002/anie.200704195 18046687

[153] T. Moyal , H. P. Hemantha , P. Siman , M. Refua , A. Brik , Chem. Sci. 2013, 4, 2496–2501.

[154] R. E. Thompson , X. Liu , N. Alonso-García , P. J. B. Pereira , K. A. Jolliffe , R. J. Payne , J. Am. Chem. Soc. 2014, 136, 8161–8164. 24873761 10.1021/ja502806r

[155] Q.-Y. Guo , L.-H. Zhang , C. Zuo , D.-L. Huang , Z. A. Wang , J.-S. Zheng , C.-L. Tian , Protein Cell 2019, 10, 211–216. 29679235 10.1007/s13238-018-0536-5 PMC6338619

[156] K. A. Kumar , L. Spasser , S. Ohayon , L. A. Erlich , A. Brik , Bioconjugate Chem. 2011, 22, 137–143. 10.1021/bc1004735 21235224

[157a] M. Murakami , T. Kiuchi , M. Nishihara , K. Tezuka , R. Okamoto , M. Izumi , Y. Kajihara , Sci. Adv. 2016, 2, e1500678; 26824070 10.1126/sciadv.1500678 PMC4730857

[157b] C. G. F. Graf , C. Schulz , M. Schmälzlein , C. Heinlein , M. Mönnich , L. Perkams , M. Püttner , I. Boos , M. Hessefort , J. N. Lombana Sanchez , M. Weyand , C. Steegborn , B. Breiden , K. Ross , G. Schwarzmann , K. Sandhoff , C. Unverzagt , Angew. Chem. Int. Ed. 2017, 56, 5252–5257; 10.1002/anie.201701362 28378443

[158] M. Murakami , R. Okamoto , M. Izumi , Y. Kajihara , Angew. Chem. Int. Ed. 2012, 51, 3567–3572; 10.1002/anie.201109034 22307754

[159a] M. Msallam , H. Sun , R. Meledin , P. Franz , A. Brik , Chem. Sci. 2020, 11, 5526–5531; 32874495 10.1039/c9sc06300e PMC7446725

[159b] M. Haj-Yahya , P. Gopinath , K. Rajasekhar , H. Mirbaha , M. I. Diamond , H. A. Lashuel , Angew. Chem. Int. Ed. 2020, 59, 4059–4067; 10.1002/anie.201913001 PMC7065254 31863676

[160] V. Agouridas , O. El Mahdi , M. Cargoët , O. Melnyk , Bioorg. Med. Chem. 2017, 25, 4938–4945. 28578993 10.1016/j.bmc.2017.05.050

